# Competition manipulation in international sport federations’ regulations: a legal synopsis

**DOI:** 10.1007/s40318-022-00210-9

**Published:** 2022-03-07

**Authors:** S. Kuwelker, M. Diaconu, A. Kuhn

**Affiliations:** Faculty of Law, University of Neuchatel, Neuchatel, Switzerland

**Keywords:** Competition manipulation (definition of), Match fixing, Betting, International federations, Reporting obligations, Aggravating and mitigating factors, Sanctions

## Abstract

Manipulation of competitions has long plagued the sport industry, affecting almost every sport over time. While sharing certain common features, the regulatory provisions and procedural responses to this phenomenon by international federations (IFs), sports’ governing bodies, vary on many aspects, including the definition of the specific offence of “competition manipulation” itself, scope of application, especially in relationship to betting, categories of participants, *mens rea* elements such as recklessness and negligent behaviour, reporting obligations, aggravating and mitigating factors, and applicable sanctions across sports and within a sport/discipline. More nuanced items within internal disciplinary procedure also vary across federations, such as standard of proof and evidence. The purpose of this study is to offer a comparative synopsis of the regulations of 43 IFs governing Olympic and certain non-Olympic sports, to provide a critical overview of specific aspects of the above mentioned factors in the regulations and to identify areas of improvement for the future.

## Introduction

### Context and relevance of study

Manipulation of competitions has long plagued the sport industry, affecting almost every sport over time,^1^ propelled in particular by certain factors such as volume of bets and quantum of money involved, inconspicuous nature of certain leagues or matches, advent of the internet, ineffective state and other legislation and, most recently, spurred on by pandemic-related economic effects.^2^

For manipulation offences in sport, primary action, whether or not in parallel with state authorities, is ordinarily initiated by the respective governing body in the sport (“*sport justice*”) against an actor engaging in such an offence.^3^ Initially, manipulation was an offence grouped with general corruption within codes of conduct or ethics, if at all—no consistent approach across federations was present. With the increase in prevalence and of its profile as a threat to integrity, as well as endeavours such as the IOC’s issued studies,^4^ and 2015 Olympic Movement Code on Prevention of the Manipulation of Competitions (“IOC 2016 Code”) serving as a model set of regulations compliant with leading international standards codified within the Council of Europe’s Macolin Convention,^5^ a number of international federations (“IF”s), adopted dedicated provisions or the IOC 2016 Code itself as a whole, such provisions continuing to apply to date.

Studying these IF’s regulations and trends across them thus assumes immense importance, in no small part as their frequent application in the first instance often results in severe consequences in long-term ineligibility for athletes, often final or not appealed from, as seen below. The specificity of drafting within provisions has important implications, including for certainty of defining an offence and its elements (for instance, including reporting obligations or negligent behaviour); types and consistency in awarding sanctions across sports and within a sport/discipline for the same offence; as well as for more nuanced items within procedure such as evidence, notoriously problematic due to the clandestine nature of acts and limited investigative ability of sporting bodies compared to state bodies. This in turn is regularly seen to have implications within adjudication on elements such as standard of proof applicable, also increasingly codified, and when the burden of proof might shift from one party to another.

### Scope

This study is limited to a review of IF regulations, and specifically the definition of manipulation therein, of all IFs with disciplines currently within the Olympic or Paralympic Games (a sub-set of all the federations recognized by the International Olympic Committee (“IOC”)) as well as, for completeness and based on prevalence of relevant offences, a few additional bodies’ regulations, international and regional.^6^ Definitions of ancillary, but connected offences usually found defined together with the “core” offence of manipulation in applicable regulations, including (illegal) betting, (dealing in) insider information, (engaging in) corrupt conduct, failing to report and cooperate are briefly looked at, without particulars of specific regulations applicable and regulating betting or reporting regulations, where present, for example.

Cumulatively, 43 IFs (Olympic/Paralympic and Non-Olympic),^7^ 1 regional governing body’s regulations (Union of European Football Associations, “UEFA”) and certain other miscellaneous regulations such as the IOC 2016 Code and Tokyo 2020 Regulations are studied. All graphs include these 43 IFs studied. The study takes into account specific decisions issued by IF internal adjudicatory processes where relevant.

Each sport, IF, abbreviation for each IF, respective relevant applicable regulation (and provision therein, if needed), and its source are systematically listed in the Annex to this article. The study below looks at IF regulations as effective in December 2020.^8^

### Analysis method

Each IF’s regulations (both specific to manipulation but also provisions from general procedure as would be applicable to manipulation offences) were assimilated and then comparatively analysed for elements within the definition (Part II below), parallel offences (Part III), sanctions (Part IV) and particulars of the specifically applicable dispute resolution processes (Part V). Statistical trends through noting common factors across these elements are represented as graphs to assess common features in how governing IFs regulations treat the same offences.

## Definition of competition manipulation by International Sports Federations

### Existence of specific regulations for the offence of manipulation

**Fig. 1:**
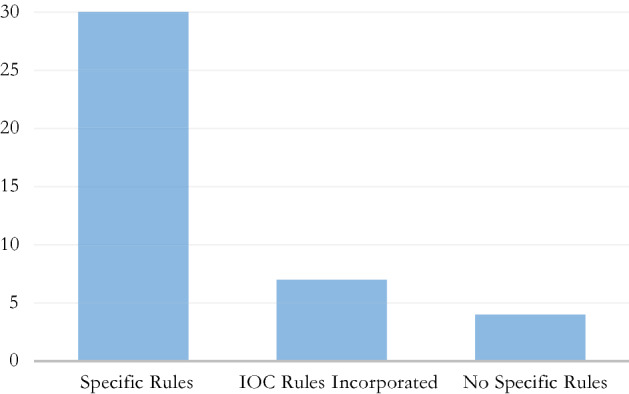
IFs with/without specific rules against manipulation of competitions

The vast majority of IFs studied have specific regulations on the manipulation of sports competitions even if, for some of them, these specific rules are limited to a few behaviours and provide that, for the rest, the IOC’s 2016 Code applies (Fig. 1). Federations that do not have specific dedicated regulations of their own generally refer to the IOC’s 2016 Code, by incorporation or through adoption of its provisions verbatim either in entirety or in part (ICF,^9^ FIG,^10^ IHF,^11^ ISSF,^12^ WCF,^13^ WBSC^14^ and WS^15^).

Only four federations do not provide for any specific rules in terms of the manipulation of sports competitions (IBSF, FIL, WDSF and IFSC) – which does not mean they do not punish match fixing categorically. Of these, one (FIL^16^) nevertheless incorporates the IOC 2016 Code for limited purposes, another (IFSC^17^) makes a reference to the IOC 2016 Code on its website as part of consent terms for athlete participation, and a third one includes certain provisions in its code of ethics (WDSF^18^).

Most IF regulations defer to applicable rules at a “major event” (as defined),^19^ such as any edition of the Olympic or Paralympic Games or a specific sport’s world cup, for governance of offences occurring at that sport. For example, at the Tokyo 2020 summer Olympic and Paralympic Games of June 2021, the issued set of “ethics” incorporated in their entirety the IOC 2016 Code,^20^ this code having been first applied at the games in Rio de Janeiro in 2016.^21^ Thus, a manipulation offence would be treated under definitions provided by the code, as well as procedure specified therein.

### Sanctionable behaviour: the “manipulation”

At the outset, it is important to note that terms such as “manipulation”, “match fixing” and “competition fixing” are often used interchangeably at the international level both in legislation and academically,^22^ and this further differs at the national level.^23^

Generally, it has been noted by several authors,^24^ but also in jurisprudence at the federation level as well as the CAS level that many different behaviours might be brought under the term “manipulation”, both *ratione materiae* and *personae*,^25^ including conduct which might not otherwise be illegal,^26^ or simply raise presumption of commission of an offence.^27^ Across IFs studied, the term “manipulation” is widely used alongside other offences, with two basic types of definitions of structures for the specific offence, whether or not termed “manipulation” can be observed, while some, and notably prominent, federations, maintain unique definitions.

The *first* broad type reflects wording in the IOC 2016 Code^28^ and the Macolin Convention,^29^ making an offence any act or omission involving (as well as complicity—aiding, abetting, encouraging, conspiring or attempting an act which could “culminate” in an offence) the alteration of the course or result of a sporting competition or a part thereof, to remove whole or part of its unpredictable nature, whether or not for or any benefit (as defined) to the actor, or to a third party, and whether or not done intentionally, negligently or fraudulently.^30^ Factors *not* relevant to this determination are usually listed including participation/attendance in the same event, outcome of event, nature of such outcome, receipt of consideration, effect on an actor’s performance and violation of any technical rules.^31^

The *second* type of definition which many federations have typically exist within older policies encompassing all types of conduct or ethics within which manipulation can be brought, even if framed after 2016.^32^ Finally, there exist miscellaneous definitions, which are important to mention as they are notably present among IFs which see a large number of cases, such as FIFA^33^ and ITF,^34^ certain others which have high profile,^35^ and new federations such as WDSF.^36^ In case of the ITF’s Tennis Anti-Corruption Program, 2020 (“TACP, 2020”), the manipulation offences are laid down as instances of common occurrences within the sport of tennis based on how matches are often manipulated.^37^

#### Active/passive

Regulations studied contain both active as well as passive manipulation of sports competitions. Moreover, 39 of the 43 federations include omission within punishable behaviour.^38^ The IOC 2016 Code also has language that includes this which has then been adopted by IFs who have incorporated the code or adopted similar language.^39^

In certain instances, common language is used across disciplinary offences or other violations to include within all of them the omission or passive contributions, bringing within its ambit aiding, abetting, other complicit, encouraging, inciting, inducing, assisting or concealing behaviour.^40^

#### Intention/negligence

An intentional act is behaviour committed with conscience and will; conversely, negligent behaviour is committed without conscience and/or without will, while nevertheless remaining at fault through culpable improvidence that can be blamed on the perpetrator.^41^

**Fig. 2:**
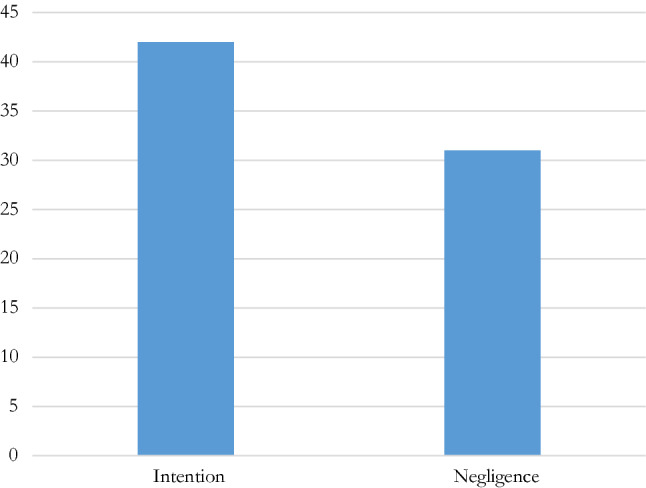
Subjective conditions in IF regulations

If intention is provided for by all the IFs that have regulated the manipulation of competitions (the only unclear situation being the IFSC^42^), only some of them also incriminate negligent behaviours, either explicitly or through other texts (such as a code of ethics, for example) extending the scope of application to negligence (Fig. 2).^43^ Among the IFs that do not expressly provide for negligence, some nevertheless provide for the punishability of negligence when certain conditions are met, such as a serious damage to the reputation of the sport or the concerned federation (IBSF^44^), or by stating, for instance, that “*ignorance*” or “*having made a mistake*” are not a defence.^45^

#### Result of competition/other parts of the event/course of the competition

Save for one IF (IFSC) where the applicable provisions are general ones not defining manipulation,^46^ all applicable federation regulations contain language which covers offences related to manipulation which affect not only the final result of the event, competition or other activity participated in or bet on, but also the course or a component of such result of events, components of events or competitions, whether affected or not.^47^

#### Financial/non-financial purpose and definition of “benefit”

The question arising here is whether the regulations aim to punish only those acts of manipulation that produce financial results or whether the punishability is extended to acts without immediate financial consequences. These include, for example, acts committed with the sole aim of obtaining a qualification or setting a record.^48^Certain instruments, such as the Macolin Convention bring intangible benefits such as advancing in competition within “manipulation”.^49^ Yet, there remains is varying opinion on criminalization of acts such as tactical losses.^50^

**Fig. 3:**
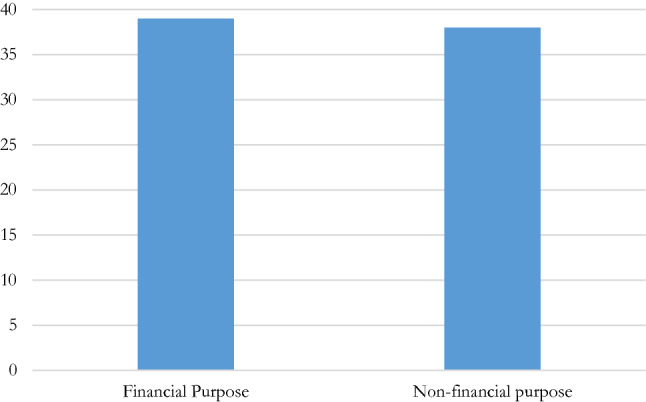
Purpose of manipulation—financial and non-financial

The study of IF regulations shows that both financial and non-financial objectives are in IF regulations’ sights (usually present in the definition of “benefit”; Fig. 3). This aspect is usually a component of the definition of “benefit”. Many IFs, particularly those having incorporated the IOC 2016 Code,^51^ have definitions including the elements of (i) the direct or indirect (ii) receipt or provision of (iii) *money or the equivalent*.^52^ Even those IFs which do not adopt the IOC 2016 Code definition strictly might still use this same definition of benefit.^53^

IFs which structure their regulations more uniquely, including those whose regulations are prominently applied, may (or may not) have alternative ways to define and apply the term “benefit”. FIFA, for instance, does not include the element of “benefit” within its definition of manipulation at all,^54^ while the ITF’s TACP, 2020 defines the more limited term “Consideration”,^55^ and ICC uses “Reward”.^56^

#### Participants, connected persons and beneficiaries (for own/for others)

Across the IFs, either the term “Participants”,^57^ “Connected Persons”^58^ or alternative terms^59^ might be used for the purposes of limiting which persons (and sometimes bodies) might be brought under the scope of the respective regulation, or could commit an offence thereunder. Some use a combination thereof, where the definition of a “Participant” will include that of a “Connected Person”, but be a sub-set of “Persons” as defined, which in turn might be a sub-set then of a wider term encompassing additional persons who could commit manipulation (or other integrity offences).^60^

The actors which might be brought under manipulation regulations or definitions vary across IFs; whether or not an exhaustive list of entities to who the regulations are applicable, and might extend from parties ranging from continental and member federations to individual athletes, judges/referees right up to anyone authorized to “co-operate, collaborate or participate” in a sport’s activities.^61^ Parties that aid, abet, encourage conspiring or contribute by their behaviour are also usually included within the scope of application.^62^ Finally, certain IFs have extended possibility to bring additional persons within the ambit of their provisions, such as the ICC’s Excluded Persons Policy, 2021.^63^

Finally, *ratione personae*, through adjudicated IF decisions and on appeal, have been seen to include a range of actors as noted by authors before.^64^ This could include both natural and legal persons, with clubs, often held strictly liable for acts of their personnel or representatives, as seen most recently in the case of Kenyan club Zoo FC^65^ and confirmed in other cases by CAS awards on appeal.^66^ This distinction/categorization is also used in certain regulations to vary which sanctions are awarded for the same offence.^67^

These actors, i.e. those to be proceeded against committing prohibited acts, might, or might not be “beneficiaries” of such conduct. Thus, as regards the beneficiaries of the manipulation, the question arises as to whether the act is punishable only if it brings a benefit to the perpetrator (as defined) or if it is also punishable when it is committed for the benefit of a third party. Such distinction is either found in the within the definition of the term “manipulation” itself,^68^ or through the language of the provision implicitly.^69^

**Fig. 4:**
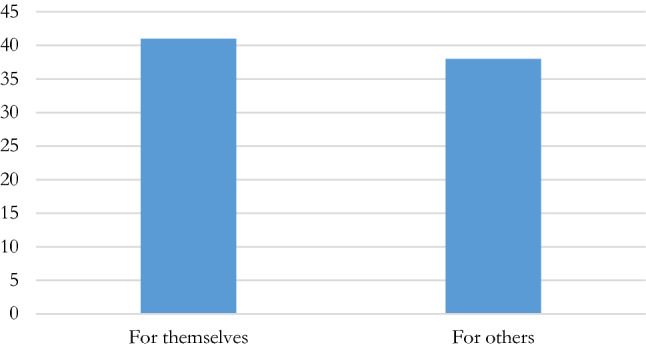
Beneficiaries of manipulations—self and/or others

Here again, the intention of most IFs is clearly to target both situations in their regulations (Fig. 4).

Connected to the definition of a “Participant”, and relevant to where/the jurisdiction in which manipulation offences take place, is the definition of what type of situation such regulations come into play. Certain IFs bring all types of activities within their purview,^70^ while others limit operations of manipulation specific regulations only to certain “Competitions”.^71^ Finally, as also mentioned above many IFs make deference to major event rules for manipulation offences there.^72^

#### Relationship to, and definition of “betting”

**Fig. 5:**
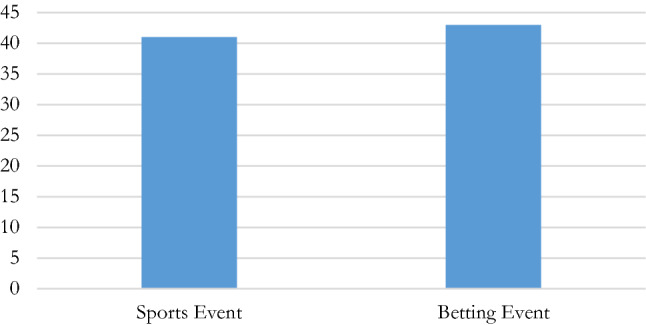
Inclusion of sporting and/or betting events

It is observed that most IFs punish manipulation-related offences in both betting and non-betting sporting contexts (Fig. 5),^73^ and usually, betting-related offences are independently defined within the same set of regulations,^74^ with the act of betting itself being an independent defined term, whether termed “bets”, “betting”, “sports betting” or “wager”.^75^ In only few instances, betting specific regulations are present without there being a corresponding wider provision to define or sanction all other forms of manipulation.^76^

### Other offences related to the manipulation of a sport competition

Under the provisions of regulations applicable to manipulation offences, most federations also define connected, overlapping or other corruption-related offences or sanctionable behaviour. In some cases, it is an overarching previous provision found within applicable regulations such as a code of ethics, prior to a specific set of regulations on competition manipulation being issued. These provisions remain most relevant in situation where no independent manipulation provision is yet defined.^77^ These could include behaviour under “bringing the sport into disrepute”, “serious misconduct”, and similarly worded behaviour.

Very notably, ITF, under the TACP and the ITF Constitution brings all connected conduct under a robust definition termed “Corruption Offences” which includes as well all the ancillary sanctionable conduct described below. Article D.1 of the TACP reads:

“*Corruption Offences: …d. No Covered Person shall, directly or indirectly, contrive the outcome, or any other aspect, of any Event. e. No Covered Person shall, directly or indirectly, facilitate any Player to not use his or her best efforts in any Event. ****f. No Covered Person shall, directly or indirectly, receive any money, benefit or Consideration on the basis of not giving their best efforts in any Event and/or negatively influencing another Player’s best efforts in any Event.**** g. No Covered Person shall, directly or indirectly, offer or provide any money, benefit or Consideration to any other Covered Person with the intention of negatively influencing a Player’s best efforts in any Event. …*”^78^

Usually, the offences of *betting* on competitions defined to be of a specific interest to the subject of the regulation (see above), *corruption* or *corrupt conduct* through the acceptance of a defined set of “benefits” whether or not connected to manipulation, and (dealing in—including using, disclosing or receiving any benefit in connection with) *insider information* are separately defined, over and above the above described offence of competition manipulation. To note, is the distinction made between betting in general and the specification of only certain kinds of betting as prohibited.^79^ Certain federations also bring under the same regulations the prohibition of receipt of gifts or benefits by officials, often above a certain value.^80^ Finally, *failure to report*, *disclose and cooperate with investigations* are also independently defined as a related offence across many federation regulations.^81^

In some instances, the offence might be specific to the sport—a relevant recent example is WDSF, where the margin of subjectivity in awarding scores across disciplines is policed specifically to curb the ability for manipulation to occur, through a common applicable code of ethics, with an independent provision within the code prohibiting direct or indirect influence of the course or result of a competition as well.^82^ Another example of specific provisions is the prohibition from participation in events organized by betting operators by the ISU.^83^

Finally, it is worth making note of the structure under UEFA’s regulations due to the sheer number of cases adjudicated under their rules as applicable to clubs.^84^ Independent provisions remain present for other actors,^85^ with connected liability for clubs.^86^ Before 2007, manipulation was proceeded against under common disciplinary rules,^87^ where a residuary provision captured all offences not specifically defined.^88^ Determined to not see clubs involved in manipulation participating in the League without consequence,^89^ since 2007, UEFA tied eligibility to prior involvement in fixing introducing a two-stage process: a primary administrative/eligibility measure, excluded a club for a single season of competitions;^90^ and a secondary disciplinary/sanctionary measure, which has no maximum duration in sanction, the primary being awarded impact on the latter.^91^

## Sanctions

### IOC’s sanctioning guidelines

To achieve some degree of harmonization among the very disparate IF regulations, the IOC has published in 2018 Guidelines for Sports Organizations on the Sanctioning of Competition Manipulation (the “IOC Sanctioning Guidelines”).^92^ This document proposes a coordinated approach on key aspects, such as factors which influence sanctions for match fixing (aggravating and mitigating), and importantly, on the level of sanctions for four key offences: betting, manipulation of sport competitions and corrupt conduct, inside information and failure to report and/or to cooperate.

For example, for the “core” offence of manipulation of sport competitions and corrupt conduct,^93^ the IOC Sanctioning Guidelines recommend a sanction of “*approx. 4 years ban and fine*” for the betting-related offence, and “*approx. 2 years ban and fine*” for the non-betting-related offence. Also, under the IOC Sanctioning Guidelines, recommended aggravating/ mitigating factors globally to consider are: (1) whether the Participant is betting on a competition she/he is participating in; (2) the number and size of the bets; and (3) addiction to betting or other specific personal circumstances.^94^

Another offence considered in the IOC Sanctioning Guidelines is the failure to cooperate, which has two faces: (1) failure to report, meaning that the participant has failed to report, at the first available opportunity, full details of any approaches or invitations received by himself or by another participant to engage in conduct or incidents that could amount to match fixing (Article 2.5 of the IOC 2016 Code), and (2) failure to cooperate with authorities during the investigation, including obstructing such investigation (Article 2.6 of the IOC 2016 Code). For failure to report or to cooperate, the IOC generally recommends a sanction of “*0–2 years ban and a fine*”; however, for obstructing the investigation (including concealing, tampering with, or destroying any relevant documentation or other information), the recommended sanction is slightly more severe, i.e. “*1-2 years ban and a fine*”. Naturally, mitigating/aggravating factors may apply.

These recommendations have had an important impact on the sanctioning regime applied by Olympic and non-Olympic IFs, many of which have chosen to incorporate them in their own regulations on match fixing. This is explained in details hereinafter.

### Types of sanctions in IF regulations

#### General sanctions

**Fig 6:**
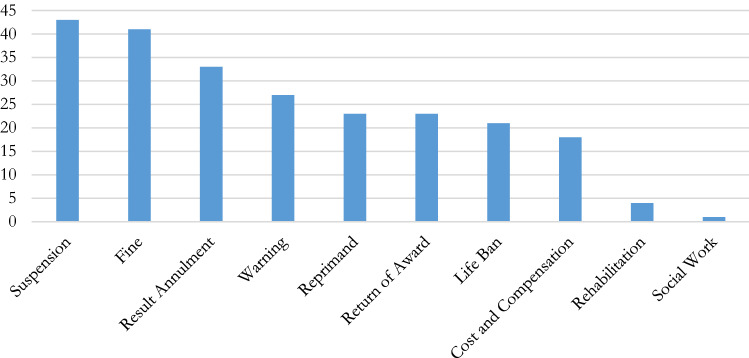
Types of sanctions and measures

The sanctions provided for by the various regulations of the international federations are of several kinds. They range from warning (with or without a period of probation) to exclusion for life, including reprimand, disqualification, return of award, fine, suspension,^95^ among others (Fig. 6). Most include provisional suspension pending investigation.^96^ Sanctions could also be categorized or listed differently for when applied to a natural person or to a legal persons/entity.^97^

*Fines* are foreseen in almost all the IFs’ regulations to sanction competition manipulation. Sometimes its amount is fixed according to the advantage received by the offender,^98^ sometimes it is limited by a maximum amount (which can vary according to whether it is imposed on a natural person or a legal entity^99^ and/or according to the seriousness of the act committed^100^) and sometimes it is unlimited/unspecified in amount.^101^ Furthermore, some regulations provide that it may be combined with other sanctions,^102^ or factors like financial hardships should be factored into the amount.^103^

Under the terms *ban, suspension or ineligibility*, one means a ban on participating in the future sporting events or administrative functions, a future that may be very limited in time but also very distant, if not unlimited.^104^ All the IFs know such suspensions that can range from temporary to perpetual, including suspensions whose duration is fixed or proportionate to the fault committed, sometimes limited in terms of both their minimum and maximum.^105^ These suspensions can also be total (in the sense that they apply to any activity related to the sport supervised by the federation)^106^ or partial (i.e. limited to certain activities supervised by the federation).^107^

*Annulment of the result*, deductions of points, return of awards and/or expulsion from the current competition, bans from venues or removal from held positions/membership from a body are also widely known sanctions in the regulations of the federations.^108^

In addition to this, some federations now award restitution, education and rehabilitation programmes, social work, reprimands (sometimes public ones), payment of procedural costs, compensation to victims, an administrative fine (independent of the fine imposed as a penalty), etc. The most “positive” measures envisaged (in the sense that they encourage the promotion of human beings rather than their stigmatization, elimination and punishment), i.e. rehabilitation programmes, education and social work, are provided for by the FINA,^109^ FIFA,^110^ IBU,^111^ WKF^112^ and UEFA.^113^

It is important to note that the type of sanction present in regulations of IFs assumes significance as it is, in most cases, deemed final even if appealed from (usually to the CAS).^114^ It is not interfered with based on the rationale of IF having expertise in the field unless found to be grossly and evidently disproportionate or irrational,^115^ the CAS having upheld a majority of decisions appealed to it from the IF dispute resolution mechanisms, including *life bans*.^116^

#### Specific sanction of life bans

The question of life bans assumes importance given this finality in awarding such bans. Recent (2021) manipulation-related decisions at the IF level have mostly seen life bans or very long, career ending bans—this is at least the case across the most prominent sports/IF from a match-fixing perspective being FIFA,^117^ ITF (5 life bans in 2021)^118^ and the ICC.^119^ BWF, for example, has also awarded life bans (to a sponsor/brand representative and three athletes for coordinating and organizing the fixing) and lengthy bans (six to 12 years with fines to other involved athletes),^120^ which are most certainly also career ending for an elite athlete, though similar bans for retired athletes, but who are likely to be involved in the sport as coaches, administrators in the future, could be argued to be insufficient as a deterrent. The most severe sanctions outside of sport (life terms, for example) are often awarded based on the gravity of a crime and only in the rarest of rare circumstances (or equivalent jurisdictional test)—thus the regularity of these in sport, if equivalent, may be debated, and has been applied with more nuance or limited to a range for other disciplinary offences, such as for doping, for example.^121^

Further, it is noted that, from a comparative perspective, life bans are similar to certain criminal or administrative sanctions, such as the interdiction to practice one’s profession or, to some extent, to lifetime detention. A lifetime ban in this context hence equals a type of “*sporting death*” of the respective person. However, unlike in most similar cases in criminal/administrative law, in sport there are no possibilities to periodically review the sentence with a view to reducing it and to reinserting the banned persons into their sport, nor is there a possibility to obtain grace, in exceptional circumstances. *Mutatis mutandis,* one may recall that, according to the jurisprudence of the European Court on Human Rights Article 3 (interdiction of torture and of inhuman or degrading treatment or punishment) of the European Convention of Human Rights must be interpreted as requiring *reducibility of the sentence*, in the sense of a *review* which allows domestic authorities to “*consider whether any changes in the life prisoner are so significant, and such progress towards rehabilitation has been made in the course of the sentence, as to mean that continued detention can no longer be justified on legitimate penological grounds*”.^122^ While potentially a stretch, this is relevant as further rights considerations are sought to be brought into IF regulations.^123^ It is thus arguable that awarding a life ban (especially as a first punishment, in the absence of a gradual scale of sanction) without any concrete possibility to reduce it in the future, raises serious issues as to its proportionality and its compatibility with fundamental principles of law.

Additionally, the issuance of life bans with this regularity requires consideration at the purpose of sanction in these contexts—whether retributive, deterrence or rehabilitation. Having noted the option of rehabilitative sanctions such as social work and education as well that a choice is made to issue life bans indicates a retributive and deterrent approach, which might serve the purpose of keeping such elements out of sport for such time to make sport clean (for the society), but not for assisting the individual. In such cases, the purpose of disciplinary sanctions in sport, could be seen to be different from that in the context of criminal law in society, otherwise.

The question of purpose however, remains independent of the consideration of proportionality, also significant as federation decisions are often final (as noted above). This made determination of the quantum an important exercise. To this end, IF bodies may consider aggravating and mitigating factors, or other guidelines, as discussed below, if so codified. Such factors may also sometimes result in the CAS concluding differently.^124^

### Aggravating and mitigating factors

**Fig. 7:**
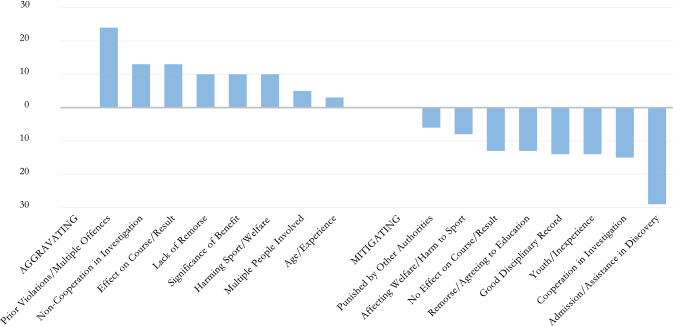
Aggravating and mitigating factors in sanctioning

Aggravating and mitigating factors are usually listed in connection with sanctions, independent of factors listed as relevant (or “not relevant”) to establishing manipulation. Usually, these include age/youth/experience/inexperience, disciplinary record/prior violations, number of breaches, significance of benefit, potential to affect course/result, whether breach was part of a broader scheme, admission of violation/cooperation/assistance/remorse^125^ (Fig. 7), and could also include, the nature of the breach(es), the degree of fault, the harm that the breach(es) has/have done to the sport, the need to deter future breaches, and other specific factors.^126^ Certain guidelines on sanctioning require, for example, considerations of “*level of responsibility or* …*equity*”.^127^ Certain federations provide for a strict tabulated range per sanctions based on how a specific manipulation offence is committed.^128^

Such factors may be seen in the regulations themselves,^129^ or even specifically applied in adjudication based on fact—the ICC’s ACU has taken into account cooperation, admission of breach, remorse, good prior record, lack of substantial damage to commercial value or public interest or matches and interestingly, the *lack of effect on the outcome of the match concerned*, in mitigating sentences.^130^ Similarly, the CAS takes into account such factors should an IF decision be overturned.^131^

It is interesting to note that certain federations make a distinction in their applicable codes between sanctions awarded for the same offences depending on factors involved (independent of the definition of the crime) such as—“fraud” (intentional breach), “fault” (whether or not intentional), “involvement” (indirect) and “attempting” (result agnostic) in their sections addressing sanctions.^132^ Similar structure can also be observed in certain IF’s sentencing guidelines applicable to all disciplinary offences specific to certain forms of cheating found in a sport such as bridge.^133^

It might be contested that the myriad factors that are present for consideration across federation guidelines result in a large amount of discretion with only vague guidance on how it is to be exercised. Comparing awards within, across sports and with other processes under law providing varied outcomes, compounds this further.^134^ Finally, it may also be observed that factors specified are often repetitive, with certain factors (non-cooperation, for instance) being offences unto themselves.

## Disciplinary procedure, dispute resolution rules and procedural issues

### Existence of separate dispute resolution procedure

**Fig. 8:**
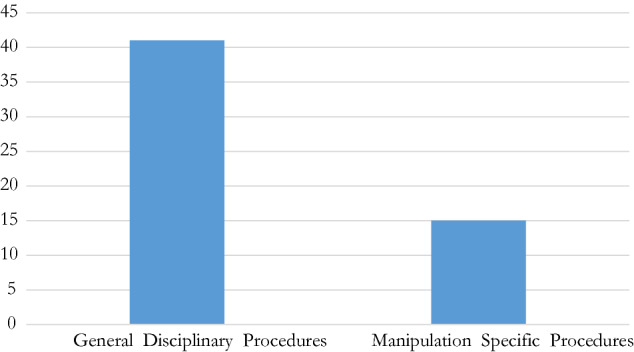
General federation disciplinary procedure and specific procedure where applied

Another question is whether there are procedures specific to an IF and, more particularly, whether there is a procedure designed to deal with cases of sports competition manipulation separately. While federation-specific procedure exists in all IFs, only some of them have an independent procedure for match-fixing cases (Fig. 8). This even if some of them know certain specific details while mainly applying a common procedure to all sports offences.

### Internal and external levels of regulation

Sanctions for competition manipulation may intervene at different levels. First, as noted above, disciplinary sanctions are applied by sports bodies, according to their internal (private) sanctions system (“*sport justice*”, the sanctions being based in contract^135^). Second, sanctions, including criminal, may be applied by public authorities (“*state justice*”). Depending on the applicable national law, the latter may be of a civil, administrative/disciplinary or criminal nature.^136^ These are considered complementary.^137^ Because they are different in their nature, these sanctions may be applied simultaneously without violating the *ne bis in idem* principle.^138^

Disciplinary sanctions issued in initial proceedings by federations internally (the process looked at later in this section) may be challenged in front of the sports bodies’ internal jurisdictional bodies and in arbitration proceedings. The jurisdictional bodies created by sport associations around the world pursue the same goal: to settle disputes, to mediate and to guarantee the correct interpretation of sporting rules and regulations.^139^

At the international level, IF bodies that have issued numerous decisions on manipulation offences include FIFA’s Disciplinary Body and Appeals Body.^140^ Similarly, UEFA’s Control, Ethics and Disciplinary Body, and Appeals Body have issued numerous decisions.^141^ Both of these sports bodies together contribute to more than 20 of around 30 to 40 cases on manipulation before the CAS (as had arisen as of the date of this study), with awards on UEFA club eligibility forming a further majority among those.^142^

Outside of these, notable federations which regularly issue internal decisions include the ITF through the International Tennis Integrity Agency (“ITIA”) which administers the TACP, 2020. At the time of study,  the ITIA lists 23 instances of persons serving sanctions not amounting to life bans and 26 instances of persons serving life bans (one such decisions being for life bans for three persons).^143^ Tennis governing bodies^144^ overall had also had three cases appealed to the CAS where two life bans and one 5-year ineligibility awarded by tennis governing bodies were upheld.^145^

Finally, but not in the least, the ICC has pronounced four decisions through its internal disputes mechanism already on corruption and fixing offences in 2021.^146^ More recently, 2 cases have involved awarding 8 years of ineligibility each for conduct violating many provisions of the ICC Anti-Corruption Code, including dealing in insider information.^147^ Certain ICC awards are also examples of awards that have been appealed to the CAS and issued alongside ongoing state proceedings, with parallel state proceedings, each factoring in the other.^148^

Among the international arbitration tribunals, the CAS was the most solicited insofar as disciplinary match-fixing proceedings are concerned, being the forum to which most federations provide for appeals from their internal disciplinary proceedings.^149^ As of the time of study, the cases dealt with by the CAS concerned five different sports.^150^ The CAS awards may be ultimately challenged in front of the Swiss Federal Tribunal (“SFT”), on the limited grounds enumerated the applicable provisions of the Swiss Federal Statute of Private International Law.^151^ As of the time of study, the SFT had not adjudicated any case related to competition manipulation in appeal from a CAS award.

### Applicable procedure within a federation

At the federation level, each international federation contains a distinct dispute resolution mechanism through which disciplinary matters are processed—i.e. a manipulation offence sought to be proceeded against by any of these federations in any discipline of any Olympic sport, has a defined channel under through which it can and will be addressed. Among these, some federations have procedures unique to manipulation offences,^152^ some have a combination of specific regulations and certain provisions applicable from common procedures or sanctions, or make specific exemptions. The rest have common processes for all disciplinary and/or other disputes within the federation^153^. Across federations, these procedures are found in dedicated regulations addressing manipulation offences, where, for instance their [above described] definitions are located. If not, the other sources of applicable provisions are spread across (and more commonly prior to the IOC’s 2016 Code) the federation’s common code of ethics, or overarching statutes/constitution.

Certain bodies have more specific reporting, investigation, prosecution and adjudication rules which are applicable.^154^ Procedural rights are laid down across most federation regulations and include the right to be informed, right to fair timely and impartial trial, right to a written defence and the right to be accompanied and/or represented. Most federation also provide which authority is to investigate and stages of progress from investigation to adjudication, for which burden and standard of proof is then described, as well as certain specific circumstances in which burden may shift.^155^

The standard of proof may range from balance of probabilities to comfortable satisfaction (sometimes specifically stated as greater than a balance of probability but less than proof beyond reasonable doubt,^156^ the same as seen at the CAS) with a prevalence of the latter. Certain rules specifically address the standard of admissibility of different types of evidence, sometimes with an illustrative list, as well as whether or not ordinary judicial rules on admissibility are applicable.

Keeping in line with this, certain IFs regulations, in line with certain CAS awards,^157^ state categorically that applicable procedure is *not* intended to be subject to or limited by requirements and legal standards applicable to criminal proceedings or employment matters but was independent and autonomous.^158^ Finally, limitation periods are also described ranging from as few as 3 years^159^ to no statute of limitations on such offences,^160^ with certain independent procedure for “smaller” offences.^161^

## Conclusions

This research has allowed us to identify the following take-home points in IF regulations as they apply to competition manipulation.

*First*, quantitatively, it was found that, as of the time of study, the vast majority of the 43 studied IFs have adopted regulations on the manipulation of sports competitions, either by producing their own detailed regulations or by reference to the IOC’s 2016 Code.

*Second*, on the regulations’ substance, it was found that applicable provisions across the offences, as at the time of being studied, remain, in their majority, robust, relevant and updated. This may also be considered an indicator of the elaborate thought given to tackling this offence relative to others—heightened attention is seen in certain sports, such as tennis or cricket, a high profile, visible sports, with many cases and high susceptibility. Even so, within regulations, the existence, or lack thereof, of a specific set of regulations, or dispute resolution mechanism assumes relevance given, as noted otherwise in awards of the CAS, or decisions of other bodies such as FIFA’s DRC that evidence within manipulation is sought to be hidden, IF investigative powers are not the same as national authorities, the offence being considered serious and the objective of preserving integrity and competition very crucial.^162^

The most important variations across sports concern basic *definitions* of the offence, and various elements thereof, as well as the quantum of *sanctions* (fines and importantly ineligibility and bans), which vary vastly. In this respect, it is important to note that the prevalence of *life bans* is significant, particularly in the context of proportionality (certain sports having a much smaller maximum ban), impact on athlete careers and the presence of alternative sanctions rooted in rehabilitation.

Yet, on a more general level, it was found that there exists disparity in detail or attention within IF regulations, which could indicate the relative significance associated by sport administration in addressing manipulation-related offences, compared to the treatment of other integrity-related offences, and in particular doping.^163^ Prior opinions have called for re-prioritization of manipulation offences, which in recent times might have slipped in public conscience in the absence of “large-scale” scandals, or increased regulation.^164^

*Third*, it was concluded that further consistency in procedural provisions across sports is desirable, aided by having uniform procedural standards applicable within a sport for various offences and across sports, given also, that sanctioning could work at various levels. This is also relevant in the light of increasing calls for protection of rights of parties and more specifically athletes, in internal IF adversarial processes.

*Last*, as a look to the near future, it is noted that newer threats within sports would need more regulatory attention—digital currency being one such example.^165^ Alongside this, growth in certain sports post new challenges. There has been a noted increase in ongoing investigations and sanctions issued across eSport,^166^ and corresponding efforts to combat manipulation therein,^167^ for example. The eSports Integrity Coalition and International Esports Federation regulations are regularly applied but have limited applicability amidst complex ownership, affiliation and governance structures across publishers, national bodies and players, as well as the different context of manipulation (such as through edoping). This makes encapsulating potential actions in a conventional definition challenging in this context.

This research has been made possible due to the support of the Swiss National Science Foundation (SNSF-100011_192497).

## Appendix: 43 Olympic and Non-Olympic International Federations Studied

**Table:**

Sr. no.	Sport and Federation Name		Applicable Regulations (as effective at the time of conclusion of the study in January 2021)
SUMMER OLYMPIC FEDERATIONS			
1.	Boxing (amateur): International Boxing Association (“**AIBA”**)	**AIBA**	AIBA Code on Prevention of Manipulation of Competitions (2018) (https://d21c25674tgiqk.cloudfront.net/2018/07/AIBA-Code-on-the-Prevention-of-the-Manipulation-of-Competitions_Final.pdf) Article 8.3.1 of the AIBA Disciplinary Code (2013) (https://d21c25674tgiqk.cloudfront.net/2015/02/AIBA-Disciplinary-Code-Adopted-July-18-2013.pdf)
2.	Badminton: Badminton World Federation (“**BWF”**)	**BWF**	Section 2.4 of the BWF Statutes, i.e. the Code on Prevention of Manipulation of Competitions (2020 or “PMC”) (https://extranet.bwfbadminton.com/docs/document-system/81/1466/1468/2.4%20PMC.pdf; the BWF Statutes are available at https://corporate.bwfbadminton.com/statutes/#1513733305001-7485aaef-d176) The BWF’s PMC also refers to the BWF’s Judicial Procedures under Section 3.1 (https://extranet.bwfbadminton.com/docs/document-system/81/1466/1469/Section%203.1%20-%20Judicial%20Procedures%20-Effective%20date%2019%20July%202020.pdf) Chapter 2—Section 2.1 of the (Code of Ethics) also defines manipulation and is subject to general procedure under Section 3.2 of the BWF’s Statutes
3.	Equestrianism: *Fédération Équestre Internationale* (“**FEI”**)	**FEI**	Appendix G (FEI Code on Prevention of Manipulation of Competitions) to FEI General Regulations (2020) (https://inside.fei.org/sites/default/files/FEI%20General%20Regulations%20effective%201%20January%202020%20-%20Final%20Version%20for%20Website%20-%20Clean.pdf)
4.	Basketball: *Fédération Internationale de Basketball Associations* (“**FIBA”**)	**FIBA**	FIBA Internal Regulations—Book 1—General Provisions (of 2020—Chapter 5: Code of Conduct) contains provisions in connection with manipulation (https://www.fiba.basketball/internal-regulations/book1/general-provisions.pdf) However, in many places e.g. certain webpages (here, for example—http://playershub.fiba.com/en/integrity/fair-play-and-betting/how-can-i-protect-myself-illegal-betting-and-match-fixing) make mention of Annexure 4 to Book 1 (All FIBA regulations otherwise are available here—https://www.fiba.basketball/documents#tab=b1506ff3-8ed5-4367-9cae-dc3e448a922f)
5.	Fencing: *Fédération Internationale d'Escrime* (“**FIE”**)	**FIE**	Appendix 1 to Chapter 12 (p. 51—Appendix 1) to the FIE Ethical Code: Betting and Anti-Corruption Rules of FIE Statutes (2015) and Chapter 7 (p. 33) being the Disciplinary Code of the FIE (https://static.fie.org/uploads/9/48811-FIE%20Statutes%20ang.pdf)
6.	Football: *Fédération Internationale de Football Associations *(*“***FIFA”**)	**FIFA**	FIFA’s Code of Ethics (2020) (https://resources.fifa.com/image/upload/fifa-code-of-ethics-2020.pdf?cloudid=upxpc0qzxqdgipiiejuj) FIFA’s Disciplinary Code (2019, referred to for sanctioning purposes Article 6.1 of the Code of Ethics, reference possible to be made under Article 52.1.d of the Disciplinary Code) (https://resources.fifa.com/image/upload/fifa-disciplinary-code-2019.pdf?cloudid=twc8yxh6fn0kjkgxhe9e)
7.	Gymnastics: *Fédération Internationale de Gymnastique* (“**FIG”**)	**FIG**	Incorporation of the IOC 2016 Code under Part 2: Article 1. c) of the FIG Code of Conduct and Article 1.c) of the FIG Code of Ethics (for Athletes, Judges and Officials, both of 2019) (https://www.gymnastics.sport/publicdir/rules/files/en_Code%20of%20Conduct%202019.pdf and https://www.gymnastics.sport/publicdir/rules/files/en_Code%20of%20Ethics%202019.pdf) Further mention of IOC 2016 Code in “Objectives” in Article 2.1 of the FIG Statutes (2019) (https://www.gymnastics.sport/publicdir/rules/files/en_Statutes%202019.pdf) Article 5 of the Code of Ethics, Infringement of this code attracts enforcement by the Disciplinary Authority under the FIG Code of Discipline (2019) (https://www.gymnastics.sport/publicdir/rules/files/en_Code%20of%20Discipline%202019.pdf)
8.	Field Hockey: *Fédération Internationale de Hockey* (“**FIH”**)	**FIH**	FIH Integrity Code (“Code”, 2018 of which Article 9 of the Code is title “Anti-Corruption Rules”) (http://www.fih.ch/media/12943893/2018-01-15-final-approved-fih-integrity-code.pdf, which superseded (see para. 1.4 on p. 1) prior specific Anti-Corruption Regulations (2017) and Article 2 of the FIH Code of Conduct Offences, in FIH Code of Conduct, 2016) Specific procedural details are present in the FIH Dispute Resolution Regulations (http://www.fih.ch/media/8997824/fih-dispute-resolution-regulations.pdf)
9.	Aquatics: *Fédération internationale de natation* (“**FINA”)**	**FINA**	FINA Rules on the Prevention of the Manipulation of Competitions (2016) (https://www.fina.org/sites/default/files/fina_rules_on_the_prevention_of_the_manipulation_of_competitions_final_30.01.2016.pdf) This is the “Implementing Provision of Article V.C.4 of the FINA Code of Ethics” (as last amended in 2015) (http://fina.org/sites/default/files/fina_codeofethics.pdf) Sanctions under C 24.8 and 24.9 of the FINA Constitution (referred to also as “FINA Rules” across some FINA documents) (https://www.fina.org/sites/default/files/_fina_constitution_19.07.2019_-_approved_by_fina_general_congress.pdf)
10.	Rowing: *Fédération Internationale des Sociétés d'Aviron* (“**FISA”**)	**FISA**	Regulation 6 on Anti-Corruption and Betting in the WR Regulations Handbook (2016) (https://integrity.worldrugby.org/resources/World_Rugby_Reg_6_EN.pdf; Reg. 6.1.3 states they “reflect” the IOC Code) General procedures in Regulation 18 Appendix 1 apply to any matters under Regulation 6 (https://www.world.rugby/handbook/regulations/reg-18/appendix-1)
11.	Volleyball and Beach Volleyball: *Fédération Internationale de Volleyball* (“**FIVB”**)	**FIVB**	The FIVB Disciplinary Regulations with Annexure A on the Prevention of Manipulations of Competitions (2020) and the FIVB Code of Ethics (2020; both available for download here—https://www.fivb.com/en/thefivb/legal)
12.	Athletics: World Athletics (until June 2019, International Amateur Athletics Federation, **“IAAF”**)	**IAAF**	IAAF Manipulation of Sport Competition Rules (“IAAF Rules”, version of 2019) (https://www.worldathletics.org/download/download?filename=88be8791-c819-4acc-826f-e37d90ada3da.pdf&urlslug=D4.2%20-%20Manipulation%20of%20Sports%20Competitions%20Rules) Rule 11 of the IAAF Rules also refers to the Reporting, Investigation and Prosecution Rules—Non-Doping (“Prosecution Rules”, version of 2019—https://www.athleticsintegrity.org/downloads/pdfs/know-the-rules/en/IAAF-AIU-Reporting-Investigation-and-Prosecution-Rules-1Jan2019-Final-18.12.2018.pdf) and to the Disciplinary Tribunal Rules (2019—https://www.athleticsintegrity.org/downloads/pdfs/know-the-rules/en/IAAF-DisciplinaryTribunalRules-1Jan2019-Final-18.12.2018.pdf)
13.	Canoeing: International Canoe Federation (“**ICF**”)	**ICF**	Incorporation of the IOC’s 2015 Code under Bylaw 4 under Bylaws to Article 2 of ICF Statutes (2019) (https://www.canoeicf.com/sites/default/files/icf_statutes_2019.pdf) ICF Statutes’ Disciplinary Measures (Article 42) and Disciplinary Processes (Article 43) are provided in Chapter IV of the Statutes (pp. 40 and 42, respectively)
14.	Climbing: International Federation of Sport Climbing (“**IFSC”**)	**IFSC**	Objectives under Article 5 bis of the IFSC Statutes (2019) include fighting manipulation and irregular and illegal betting and gambling and Bylaw to Article 10 defined (p. 16 of the IFSC Statutes defines violation of illegal and irregular betting but not manipulation) (https://cdn.ifsc-climbing.org/images/About/2020_IFSC_Statute_Approved.pdf) Certain procedure specified in the IFSC Disciplinary and Appeals Rules (“DAR”, 2007, updated 2019) (https://cdn.ifsc-climbing.org/images/ifsc/Footer/Commissions/Disciplinary_AppealsRules2019.pdf) IFSC website provides for athletes to sign an undertaking adhering to the IOC 2016 Code (“Please note that starting in 2017, the Athletes License form includes specific reference to the Olympic Movement Code on the Prevention of the Manipulation of Competition which all athletes are required to read and sign”.) (https://www.ifsc-climbing.org/index.php/2-uncategorised/49-athletes)
15.	Golf: International Golf Federation (“**IGF”**)	**IGF**	IGF Betting and Anti-Corruption Policy (2016) (http://www.igfgolf.org/wp-content/uploads/2017/01/IGF-Betting-and-Anti-corruption101116.pdf)
16.	Handball: International Handball Federation (“**IHF”**)	**IHF**	Incorporation of the IOC 2016 Code under Articles 4 and 5 of the IHF Ethics Code (2016, p. 8) (https://www.ihf.info/sites/default/files/2019-06/0_0_Ethics%20Code_GB.pdf—Annexed as an Appendix, p. 14 of the pdf) Article 11, Enforcement is in accordance with IHF Statutes (2018) (https://archive.ihf.info/files/Uploads/NewsAttachments/0_01%20-%20Statutes_GB.pdf) Article 1 of the IHF Regulations Concerning Penalties and Fines (2017) states that its provisions are specifically applicable to “corruption” and “betting” offences (https://www.ihf.info/sites/default/files/2019-05/0_XIX%20Regulations%20Concerning%20Penalties%20and%20Fines_GB.pdf) Article 3 of the Ethics Code otherwise specifically states that “*In the event of any inconsistency between the Code and any other IHF Regulations except for the IHF Statutes, the relevant provisions of the Code shall prevail*”.
17.	Judo: International Judo Federation (“**IJF”**)	**IJF**	Article 1.2.2 (Match-fixing and Competition Manipulation) and Annexure F (Disciplinary Code, p. 153) of IJF’s Sport and Organization Rules (“SOR”, 2019) (https://78884ca60822a34fb0e6-082b8fd5551e97bc65e327988b444396.ssl.cf3.rackcdn.com/up/2019/10/IJF_Sport_and_Organisation_Rul-1570787163.pdf) Another reference to corruption is made in the Code of Ethics (2019, p. 153 of the same SOR document
18.	Surfing: International Surfing Association (“**ISA”**)	**ISA**	ISA’s Code on the Prevention of Manipulation of Competitions is located under Section 3 (Contest Rules and Procedures Part A, subpart v—General) of Chapter 2 (ISA Event Administration), under the larger ISA Rulebook (2019) (https://www.isasurf.org/wp-content/uploads/downloads/2019/06/ISA-Rulebook_-13-June-2019.pdf) Section 3.A.v (see above), refers to a specific hyperlinked code (2019) (http://www.isasurf.org/wp-content/uploads/downloads/2019/06/ISA-Rules_Prevention-of-the-Manipulation-of-Competitions.pdf)
19.	Sailing: World Sailing (until December 2015, International Sailing Federation; “**ISAF”**)	**ISAF**	Appendix 5 (Regulation 37)—Betting and Anti-Corruption Code of the (World Sailing Regulations, “Regulations”, 2020) and Appendix 6 (Regulation 25)—Disciplinary Appeals and Review Code (of the Regulations (combined document available here—https://www.sailing.org/tools/documents/2020RegulationsClean-[26381].pdf) Other sanctions from the WS Code of Ethics (2019) might also be imposed (https://www.sailing.org/tools/documents/2019CodeofEthicsPostMidYear-[25097].pdf)
20.	Shooting: International Shooting Sport Federation (“**ISSF”**)	**ISSF**	ISSF Code of Ethics (“Code”, Article 3.12.3.5, Annex CE) of the ISSF General Regulations (2020), governs manipulation, the preamble stating that “*ISSF with these regulations also implements the new IOC Olympic Movement Code on the Prevention of the Manipulation of Competitions. The Definitions used in version 2016 of such IOC Code also apply to the following rules*” (https://www.issf-sports.org/getfile.aspx?mod=docf&pane=1&inst=465&file=ISSF%20Code%20of%20Ethics.pdf)
21.	Tennis: International Tennis Federation (“**ITF”**)	**ITF**	Tennis Anti-Corruption Program (2020) and Rule 43 of the ITF Constitution 2020 which mandates compliance with the IOC 2016 Code (https://www.itftennis.com/media/2703/2020-tennis-anti-corruption-program-english.pdf and www.teniisintegrityunit.com; https://www.itftennis.com/media/2431/the-constitution-of-the-itf-2020-english.pdf, respectively)
22.	Table Tennis: International Table Tennis Federation (“**ITTF”**)	**ITTF**	Code on the Prevention of Manipulation of Competitions in the Code of Ethics (“Code”, in Chapter 6) of the ITTF Handbook (2020) (https://www.ittf.com/wp-content/uploads/2020/04/2020ITTFHandbook_v1.pdf)
23.	Triathlon: International Triathlon Union (“**ITU”**)	**ITU**	ITU Rules on the Prevention of Manipulation of Competitions (“Rules”, 2016) incorporate by reference the IOC 2016 Code and make mention of the Rules and IOC 2016 Code being in compliance with the Macolin Convention under Rule 1.3. (https://www.triathlon.org/uploads/docs/ituafl_rules-preventing-manipulation.pdf) ITU Disciplinary Rules (2016) under Article 13 contain details on matters such as admissibility of evidence (https://www.triathlon.org/uploads/docs/ITU_Disciplinary_Procedures_Rules_20160708.pdf)
24.	Weightlifting: International Weightlifting Federation (“**IWF”**)	**IWF**	IWF Guidelines: Competition Fixing (2015) (https://www.iwf.net/wp-content/uploads/downloads/2015/11/IWF-Guidelines_Competition-Fixing.pdf) Certain sanctions possible to apply under IWF’s Constitution and By-Laws (2017, p. 21) (https://www.iwf.net/wp-content/uploads/downloads/2017/04/IWF_ConstitutionBy-Laws__2017_pdf)
25.	Cycling: *Union Cycliste Internationale* (“**UCI”**)	**UCI**	Article 8.1 (Manipulation of Cycling Events) and corresponding Appendix 2 to Code of Ethics (https://www.uci.org/docs/default-source/rules-and-regulations/uci-code-of-ethics.pdf) governs manipulation (except betting) Article 1.1.088 of the UCI Cycling Regulations which defines betting (version 2020, p. 23) (https://www.uci.org/docs/default-source/rules-and-regulations/part-i-general-organisation/1-gen-20200612-e.pdf)
26.	Modern Pentathlon: *Union Internationale de Pentathlon Moderne* (“**UIPM”**)	**UIPM**	Betting and Anti-Corruption Rules (2018, being Part V of the UIPM Constitutional Book) (https://www.uipmworld.org/sites/default/files/betting_and_anti_corruption_rules_uipm_2018.pdf) UIPM Code of Ethics (2018) lays down procedural guidelines, including “Procedural Rules” (Annex 1 to the Code of Ethics) (https://www.uipmworld.org/sites/default/files/code_of_ethics_uipm_2018.pdf)
27.	Wrestling: United World Wrestling (“**UWW”**)	**UWW**	Incorporation of IOC 2016 Code under Article 1 in the Code of Ethics (2018, p. 10) (https://unitedworldwrestling.org/sites/default/files/2018-12/8_code_of_ethics_eng.pdf) Additional reporting guidelines (not obligatory) under “Reporting acts against the integrity of the Sport” (https://unitedworldwrestling.org/sites/default/files/2020-01/3.5_policy_reporting_acts_vs_integrity.pdf) Procedure under Disciplinary Code and Dispute Resolution Regulations (effective 2018) (https://unitedworldwrestling.org/sites/default/files/2019-09/disciplinary_proced_dispute_resolution_regulations_june2019_final_eng.pdf)
28.	Archery: World Archery (“**WA”**)	**WA**	Appendix 9 to Book 1 of the Constitution and Procedures (Betting and Anti-Corruption) (version of October 2020) (p. 43—https://rulebook.worldarchery.org/PDF/Official/2020-10-01/EN-Book1.pdf)
29.	Baseball and Softball: World Baseball and Softball Confederation (“**WBSC**”)	**WBSC**	Application/adoption of the IOC 2016 Code through Article 5.2 of the WBSC Code of Ethics (“Code”, 2017) Specifically, within the Code, it is stated that the By-laws regarding Sports Betting (2017) must be applied for manipulation (presumably in addition to the Code). These regulations are worded identically to the IOC 2016 Code, with additional obligations in connection with illegal betting (and gambling—see Chapter II). Disciplinary Procedure is under Chapter III of the Code. All these provisions echo the IOC 2016 Code Presumably, the Disciplinary Regulations (2017) would also apply in addition, for procedure and sanctions. Certain sanctions also in Code. (https://www.wbsc.org/organisation/statutes)
30.	Dance Sport (Breaking): World Dance Sport Federation (“**WDSF”**)	**WDSF**	WDSF Code of Ethics (https://www.worlddancesport.org/Rule/Athlete/Code_of_Ethics#:~:text=The%20values%20and%20principles%20written,in%20particular%20its%20fundamental%20principles) Disciplinary processes under WDSF Disciplinary Council Code (https://www.worlddancesport.org/Rule/WDSF/Regulations/WDSF_Disciplinary_Council_Code) Further judicial procedure under the WDSF Internal Dispute Resolution Code (https://www.worlddancesport.org/rule/all)
31.	Karate: World Karate Federation (“**WKF”**)	**WK**	WKF Code on the Prevention of Manipulation of Competitions (2016) (https://www.wkf.net/pdf/manipulation-of-competitions-pdf-en-236.pdf) Procedural provisions present in the WKF Disciplinary and Ethics Code (Article 3.1.3 and 3.6) (https://www.wkf.net/pdf/wkf-disciplinary-and-ethics-code_15march2016-pdf-eng.pdf)
32.	Rugby Union: World Rugby (“**WR”**)	**WR**	Appendix 8—Bylaws to Article 59—FISA Code of Ethics (“Code”, p. 143) and Appendix 9—Bylaws to Article 60—Manipulation of Competition and Betting (“Manipulation Bylaws” p. 149) both in Part V—Integrity of the Sport (p. 34) and Part VI—Judicial Provisions (p. 35; all within the FISA Rule Book, of 2017) (http://www.worldrowing.com/mm//Document/General/General/13/58/39/FISArulebookEN2019web_Neutral.pdf) govern manipulation.)
33.	Taekwondo: World Taekwondo (“**WT”**)	**WT**	WT’s Bylaws on Betting and Anti-Corruption (2012, “Bylaws”) (http://www.worldtaekwondo.org/viewer_pdf/external/pdfjs-2.1.266-dist/web/viewer.html?file=http://www.worldtaekwondo.org/wp-content/uploads/2016/10/WTF-Bylaws-on-Betting-and-Anti-Corruption.pdf)
34.	Skateboarding: World Skate (“**WS**”)	**WS**	WS Code of Conduct and Code of Ethics (2019—p. 9, Section I, subsection 6.A.4 and 6.A.7 (making reference to application of IOC 2016 Code) and p. 17, Section II, subsection 2.C—definitions) Certain procedure (prosecution and investigation) is contained in the WS Bylaws under para. 8, Section C.6 (2019) (http://www.worldskate.org/about/documents/category/304-world-skate-charters.html)
**WINTER OLYMPIC FEDERATIONS**			
1.	Luge: *Fédération Internationale de Luge de Course* (“**FIL”**)	**FIL**	Part II (Specification of the Guidelines for Action), and Article 2 of the FIL Ethics Code (2019); as well, the FIL website generally (https://www.fil-luge.org/cdn/uploads/fil-ethik-code-englisch.pdf) and on the FIL website in general (https://www.fil-luge.org/en/anti-doping-fairplay/prevention-of-competition-manipulation; see also and on the FIL website in general (https://www.fil-luge.org/en/anti-doping-fairplay/prevention-of-competition-manipulation) Procedure and sanctions are present FIL Statutes (2019) and the Law and Procedure Regulations specifically commencing on p. 15 (https://www.fil-luge.org/cdn/uploads/statuten-2019-komplett-english-inkl-aenderungen.pdf)
2.	Skiing and Snowboarding: *Fédération Internationale de Ski* (“**FIS”**)	**FIS**	FIS Rules on the Prevention of Manipulation of Competitions (2016) (https://assets.fis-ski.com/image/upload/v1536910200/fis-prod/assets/FIS_Rules_on_the_Prevention_of_the_Manipulation_of_Competitions.pdf)
3.	Bobsleigh and skeleton: International Bobsleigh and Skeleton Federation (“**IBSF”**)	**IBSF**	General provisions present in the Code of Ethics (effective September 2011, not strictly related to manipulation) (https://www.ibsf.org/images/documents/downloads/code_of_ethics_E_120712_IBSF.pdf) Code of Conduct to be signed by athletes (2018 version) before obtaining IBSF license contains provisions in connection with manipulation (https://www.ibsf.org/images/documents/downloads/Codes_of_Conduct/20180725_CoC_athletes_2018.pdf) Certain provisions under Statutes of the IBSF (2019) also allude to manipulation and adoption of the IOC 2016 Code (Article 3.15) (https://www.ibsf.org/images/documents/downloads/IBSF_Statutes_am-congress2019.pdf)
4.	Biathlon: International Biathlon Union (“**IBU”**)	**IBU**	Chapter C (Prevention of Manipulations of Competitions) of IBU Integrity Code (“Code”—of 2019, which is part of IBU’s constitution, applicable to all cases where violations happen after the “Effective Date” as defined 19 October 2019); this document replaced a number of previous documents (Code of Ethics, anti-doping rules etc.) (https://res.cloudinary.com/deltatre-spa-ibu/image/upload/tziqluncgelica29obn7.pdf)
5.	Ice hockey: International Ice Hockey Federation (“**IIHF”**)	**IIHF**	IIHF Code of Conduct (“Code”, 2021) (https://blob.iihf.com/iihf-media/iihfmvc/media/downloads/regulations/2021/2021-iihf-code-of-conduct.pdf) See also ancillary provisions which pre-date the above Code such as Bylaw 24.1 on Manipulation of Competitions in the IIHF Statutes and Bylaws, requiring compliance with the Code of Conduct provisions on manipulations 2018–2022 and 2021–2024 (https://blob.iihf.com/iihf-media/iihfmvc/media/downloads/statutes/2021-2024-statutes-and-bylaws-(effective-september-2021).pdf)
6.	Ice skating: International Skating Union (“**ISU”**)	**ISU**	Articles 7, 8, 11, 12, 13, 16 and 17 of the ISU Code of Ethics (“Code”, 2018) (https://www.isu.org/inside-isu/isu-communications/communications-archives/19025-isu-communication-2215/file) Disciplinary Procedure and Sanctions are provided under the ISU Constitution and General Regulations (2018, referenced in Article 16 of the Code) (https://www.isu.org/inside-isu/rules-regulations/isu-statutes-constitution-regulations-technical/17913-constitution-general-regulations-2018/file)
7.	Curling: World Curling Federation (“**WCF”**)	**WCF**	Adoption of IOC 2016 Code by incorporation through Adoption Resolution (https://s3.eu-west-1.amazonaws.com/media.worldcurling.org/media.worldcurling.org/wcf_worldcurling/2019/08/05125448/Olympic_Movement_Code_on_the_Manipulation_of_Competitions_approved_at_2017_AGA.pdf) See also reference in Article 8 of the Code of Ethics (2016) (https://s3.eu-west-1.amazonaws.com/media.worldcurling.org/media.worldcurling.org/wcf_worldcurling/2019/08/05124858/Code_of_Ethics.pdf) Procedural provisions under Disciplinary Sanctions/Escalation/Appeals Policy (2017) (https://s3.eu-west-1.amazonaws.com/media.worldcurling.org/media.worldcurling.org/wcf_worldcurling/2019/08/05125501/Disciplinary_Sanctions_Escalation_Appeal_Policy_Sept_2017.pdf; this is to be read with other policies such as an independent available Escalation Policy (2016)—https://s3.eu-west-1.amazonaws.com/media.worldcurling.org/media.worldcurling.org/wcf_worldcurling/2019/08/05125204/Escalation_Appeals_Policy.pdf)
**NON-OLYMPIC FEDERATIONS**			
1.	Cricket: International Cricket Council (“**ICC**”)	**ICC**	ICC’s Anti-Corruption Code for Participants (2018, amended 2021) and Minimum Standards for Players and Match-Officials’ Areas at International Matches (https://resources.pulse.icc-cricket.com/ICC/document/2021/01/06/a40dd941-47d0-4dec-8798-6e2885ad0fcd/ICC-Anti-Corruption-Code-amended-with-effect-from-1-Jan-2021-Board-approved-Nov-2020-.pdf; and https://icc-static-files.s3.amazonaws.com/ICC/document/2018/12/16/f3b76dad-9668-4a64-aeb8-0e8684d8e28b/-FV-Effective-from-1-December-2018-PMOA-Minimum-Standards-CEC-approved-.pdf)
2.	Bridge: World Bridge (“**WB**”)	**WB**	Article 4 of the WB Code of Ethics (2016) incorporates the IOC 2016 Code (http://www.worldbridge.org/wp-content/uploads/2016/12/WBFCodeofEthics.pdf) Sanctions under Disciplinary Code (2016) and specific Sentencing Guidelines making it contingent on nature of offence. (http://www.worldbridge.org/wp-content/uploads/2020/06/WBFDisciplinaryCode.pdf; and http://www.worldbridge.org/wp-content/uploads/2016/11/WBFSentencingGuidelines.pdf) Anti-betting Regulation (2011) defines betting differently (http://www.worldbridge.org/wp-content/uploads/2016/11/WBFAntiBettingRegulations-1.pdf)
**MISCELLANEOUS**			
1.	Football (Europe): Union of European Football Associations (“**UEFA**”)	**UEFA**	Administrative (Eligibility Related): Article 4.02 of the UEFA Champions League and Article 4.03 of the UEFA Europa League Regulations (Champions League (football)—https://documents.uefa.com/r/Regulations-of-the-UEFA-Champions-League-2020/21-Online; the sport of futsal has independent regulations and Europa League—https://documents.uefa.com/r/Regulations-of-the-UEFA-Europa-League-2020/21-Online) Disciplinary Regulations (Sanctions Related): Article 12 of UEFA Disciplinary Regulations (Edition of 2020) (https://documents.uefa.com/v/u/ZNsWJsRSmOuSS2Ql_y8~qQ)

## Footnotes

1 See the historical evolution as presented in Chappelet and Verschuuren (2019, p. 231).

2 This was addressed more recently by UNODC, IOC and INTERPOL through their issued dossier on Preventing Corruption in Sport and Manipulation of Competitions (2020); for general causes, see INTERPOL-IOC (2016) in their Handbook on Protecting Sport from Competition Manipulation, pp. 19 and 23; on the correlation to betting in specific, see Chappelet and Verschuuren (2019, pp. 431 and 432).

3 On the initial distinction of recourse in dispute process within sport as opposed to under national legislation, see, for example, Nafziger (1996). The Court of Arbitration for sport has held that “*disciplinary sanctions imposed by associations are subject to civil law and must clearly be distinguished from criminal penalties*” imposed by a state—*Johannes Eder* v. *Ski Austria*, CAS 2006/A/1102, award dated 13 November 2006 at para. 52.

4 These studies have focused on model regulations (such as the IOC 2016 Code, *infra* note 9), model criminal law provisions for nations, and include reports on countries’ criminalization based on studies of country-wise approaches, the latest study published in 2021, specific aspects within manipulation investigation at the IF and national level, such as reporting mechanisms [on these, see IOC-UNODC (2019) on Reporting Mechanisms in Sport].

5 See Preamble (clause c) of the IOC’s Olympic Movement Code on Prevention of the Manipulation of Competitions (2016, “IOC 2016 Code”); see also, Council of Europe’s Convention on the Manipulation of Sports Competitions, CETS No 215, having entered into force September 1, 2019, known as the Macolin Convention and also open for ratification to countries outside Europe.

6 This includes (1) the ICC (due to the large prevalence of manipulation offences and of high profile, within cricket); (2) WB (having had a case on manipulation within bridge, one of five sports, appealed and award issued by the CAS—*Fulvio Fantoni and Claudio Nunes* v. *European Bridge League*, CAS 2016/A/4783, award dated 10 January 2018, albeit applying European Bridge League rules, and not WB rules specifically); (3) the relevant regulations as applicable to the Tokyo 2020 Olympic and Paralympic Games—the *Code of Ethics and Other Texts: Prevention*—*Manipulation*—*Tokyo 2020* (2018b, the “Tokyo 2020 Regulations”) across sports as is common practice among major events (to supersede IF regulations which defer to such games’ rules); and (4) regionally, UEFA for the number of cases originating in those regions. A final emerging threat among international sport disciplines for manipulation is eSport, briefly addressed in Sect. 5 (Conclusion).

7 AIBA, BWF, FEI, FIBA, FIE, FIFA, FIG, FIH, FIL, FINA, FIS, FISA, FIVB, IAAF, IBSF, IBU, ICC, ICF, IFSC, IGF, IHF, IIHF, IJF, ISA, ISAF, ISSF, ISU, ITF, ITTF, ITU, IWF, UCI, UIPM, UWW, WA, WB, WBSC, WCF, WDSF, WK, WR, WT and WS—see Annex.

8 Save for ICC’s Anti-Corruption Code and other applicable regulations which were amended in January 2021, but were effective from February 2018.

9 Incorporated in entirety under Bylaw 4 of the Bylaws to Article 2 of ICF Statutes (2019).

10 Incorporation by reference under Part 2: Article 1. c) of the FIG Code of Conduct and Article 1.c) of the FIG Code of Ethics (for Athletes, Judges and Officials, both of 2019). The respective provision in both codes states that “*All points contained in…the Code*” are to be “*scrupulously observed*”. Further, under “Objectives” in Article 2.1 of the FIG Statutes (2019) an objective “*to respect the IOC Charter and the IOC Code of Ethics including its Implementing provisions, in particular …the Olympic Movement Code on the Prevention of the Manipulation of Competitions*” is stated. However, unlike other IFs, the IOC Code or its provisions are not actually reproduced in the federation’s own statutes.

11 Articles 4 and 5 of the IHF Ethics Code (2016, p. 8) incorporate the IOC 2016 Code in its entirety (Annexed as an Appendix, p. 14).

12 ISSF Code of Ethics (Article 3.12.3.5, Annex CE) of the ISSF General Regulations (2020), governs manipulation. In its preamble, it states that “*ISSF with these regulations also implements the new IOC Olympic Movement Code on the Prevention of the Manipulation of Competitions. The Definitions used in version 2016 of such IOC Code also apply to the following rules*”.

13 Incorporated through adoption, through Adoption Resolution available here; see also reference in Article 8 of the WCF Code of Ethics (2016).

14 Article 5.2 and Chapter V of the WBSC Code of Ethics 2017 make reference to the IOC 2015 Code.

15 Item 6.A.7 under Principles (Clause 6) of the WS Code of Ethics, 2019.

16 Manipulation finds mention in Part II (Specification of the Guidelines for Action), and Article 2 of the FIL Ethics Code (“FIL Ethics Code”, 2019). Article 2.7.8 of the FIL Ethics Code makes monitoring and enforcement of certain specified provisions of the IOC 2016 Code (Articles 1, 2 and 6) the mandate of the code. Further on, these Articles, covering definitions, what constitutes a violation, factors relevant and not relevant and mutual recognition provisions, among others, are extracted in Part 7 (Other Applicable Rules and Commencement). However, general provisions from “*the rules of procedure and sanctions of FIL Statutes, the FIL Law and Procedure Regulations, and the FIL Ethics Code*” also apply, as do complementary procedural provisions.

17 The following statement is found under the section on “Athletes”—“*Please note that starting in 2017, the Athletes License form includes specific reference to the Olympic Movement Code on the Prevention of the Manipulation of Competition which all athletes are required to read and sign*” (available at IFSC website (https://www.ifsc-climbing.org/index.php/2-uncategorised/49-athletes, March 29, 2021). Otherwise, IFSC’s provisions related to manipulation offences are generally found under Article 5 bis of the IFSC Statutes (2019) which states that one of the IFSC’s objectives is “*To fight against competition manipulation*” and “*To fight against irregular or illegal betting or gambling*” while the Bylaw to Article 10 (on p. 16 of the IFSC Statutes) defines the violation of “Illegal and irregular betting”, but not manipulation.

18 Under the WDSF Code of Ethics (version 1.1) as available on www.worlddancesport.org (March 29, 2021) Article 2.5, titled “Idealism” under Article 2 dealing with Fundamental Ethical Principles in Dance Sport, provides for 2 offences (Article 2.5.1 on all types of betting or wagering, and Article 2.5.2 on general manipulation).

19 See, for example, Under Rule 37.7.2 decisions taken at major events based on similar rules would be recognized by ISAF’s Betting and Anti-Corruption Code under Appendix 5 (Regulation 37) of the of the (World Sailing Regulations, “Regulations”, 2020—ISAF now being called “World Sailing”).

20 See Articles 3 and 4 of the IOC’s Tokyo 2020 Regulations.

21 See, as published on IOC’s website, article titled “*IOC publishes unprecedented olympic movement code for preventing competition manipulation*” (2015).

22 Diaconu et al. (2021) in Part 2 “Terminology”.

23 See report of the UNODC and IOC (2017, pp. 22–35) where terms such as “*bribery in sporting contests*” (United States) or “*corrupt activities relating to sporting events*” (South Africa) might also be used in state legislation.

24 See Palermo and Williams (2018, p. 1).

25 Beyond the scope of this article, see Diaconu et al. (2021) at Parts 6.1 (*Ratione Materiae*) and 6.2 (*Ratione Personae*).

26 *Vsl Pakruojo FK , Darius Jankauskas, Arnas Mikaitis, Sigitas Olberkis, Valdas Pocevicius, Alfredas Skroblas, Donatas Strockis, Diogo Gouveia Miranda, C.H. Alexandru and Taras Michailiuk* v. *Lithuanian Football Federation* (“LFF”)*,* CAS 2015/A/4351 on appeal from the Lithuanian Football Federation’s Appeals Body, dated 13 July 2016 at paras 81(i), paras 91–92 (“Pakruojo”).

27 *Eskişehirspor Kulübü* v. *UEFA,* CAS 2014/A/3628, award on appeal from UEFA, dated 2 September 2014 at paras 114–119.

28 Under Article 2.2, with the rest of the same article providing independent definitions of Betting (2.1), Corrupt Conduct (2.3), Inside Information (2.4), Failure to Report (2.5) and Failure to Cooperate (2.5) of the IOC 2016 Code.

29 Article 3 part 4 of the Macolin Convention. Note the other definitions within the Macolin Convention (e.g. on betting under Article 3 part 5) are not the same.

30 See, for example, federations like the ICF who have adopted the IOC 2016 Code in its entirely, or others such as WS (Part II.2.C, pp. 16 and 17 of its Code of Conduct and Code of Ethics, 2019) who have used verbatim the provisions within their own drafted codes; this/similar definitions are also adopted by IAAF, BWF, AIBA, UCI, FEI, FIG, FIL, IHF, FIH, IIHF, IJF, ISSF, ISU, FIVB, IWF, UWW, ISA, WKF, WCF, WB, WBSC and WS.

31 Article 2.7, IOC 2016 Code. It is important to note that in certain regulations, aggravating and mitigating factors are listed specific to only sanctioning and aggravating and mitigating factors are listed exhaustively independent of one another—see Articles 6.1.1 and 6.1.2 of the ICC Anti-Corruption Code (2019).

32 See for example, Rule 3.2 of the IGF Betting and Anti-Corruption Policy, 2016 which says “*Fixing or contriving in any way or otherwise improperly influencing, or being a party to fix or contrive in any way or otherwise improperly influence, the result, progress, outcome, conduct or any other aspect of an event or competition under the jurisdiction of the IGF. ...Ensuring or seeking to ensure the occurrence of a particular incident in an event or competition under the jurisdiction of the IGF which occurrence is to the Participant’s knowledge the subject of a Bet and for which he/she or another Person expects to receive or has received a Benefit. ...Failing in return for a Benefit (or the legitimate expectation of a Benefit, irrespective of whether such Benefit is in fact given or received) to perform to the best of one’s abilities in an event or competition under the jurisdiction of the IGF. ...Inducing, instructing, facilitating or encouraging a Participant to commit a Violation set out in this Rule (3.2*)”; FIE, FIBA, FIS, WA, ITU, WT, ISAF, WR, FISA and UIPM all have similarly worded definitions, while the ITTF, for example, has both this one and another worded like the IOC 2016 Code.

33 Article 29 of the FIFA Code of Ethics (2020). Although the language remains similar to the IOC 2016 Code, it is simplified, and structure of regulation different, notably including liability specifically for clubs, independent of definitions for “participants” or “benefits” seen below. See footnote 54 under Sect. 2.2.4 “Financial/non-financial purpose and receipt of Benefits”.

34 The ITF brings manipulation under a broader definition of “Corruption Offences” under the TACP (2020), *infra* note 78; Rule 43 of the ITF Constitution (2020) mandated compliance with the IOC 2016 Code as well.

35 See FINA’s definition under PMC 3.3 of the FINA Rules on Prevention of the Manipulation of Competitions (“PMC Rules”, 2016) being—“*Directly or indirectly contriving or attempting to contrive the outcome or any other aspect of any Competition with the goal of obtaining a Benefit for oneself or for others*.”

36 Which includes broad language on altering the course or outcome of a competition or betting related offences within its Code of Ethics (undated, version 1.1).

37 See the extracted provision from the TACP, 2021 at *infra* note 78.

38 IFs such as FINA have used language under Rule 10.2 of the FINA PMC Rules to explicitly specific that “*Knowingly assisting, covering up or otherwise being complicit in any acts or omissions of the type described in these Rules*” was also a violation under the rules.

39 ICF’s adoption of the IOC 2016 Code under ICF’s Statutes (2019)—*supra* note 9. The IOC 2016 Code under Article 2.7 includes the term “omissions” under the listed factors to not be taken into account when considering constitution of the crime, i.e. “*e. Whether or not the Participant’s effort or performance in the Competition concerned were (or could be expected to be) affected by the acts or omission in question; and f. Whether or not the result of the Competition concerned was (or could be expected to be) affected by the acts or omission in question*.” are irrelevant to the constitution of the offence and thus included within the definition of manipulation indirectly.

40 See Item 166 under the FIBA Internal Regulations—Book 1—General Provisions (of 2020—Chapter 5: Code of Conduct, contains provisions in connection with manipulation), where acts or omissions amounting to attempted violations or to any type of complicity involving a violation or attempted violation, including, without limitation, aiding, abetting, inciting, inducing, instructing, facilitating, encouraging, assisting or covering up a violation or attempted violation of the Code of Conduct also was a Disciplinary Offence.

41 For a discussion on intention, negligence and strict liability within match-fixing, see Diaconu and Kuhn (2019, p. 9). The issue of negligence in sporting disputes has been discussed across various contexts including, for instance, liability for sporting injury (more of a “civil” nature), as well as disciplinary instances such as doping violations (more akin to a sanction-worthy or even criminal offence) where strict liability has increasingly been adopted for offences committed. For the former, see, for instance, Rosenthal (2004); for the latter, see McArdle (2015), the commencement of the debate having been discussed years prior—see Rigozzi et al. (2003, p. 39).

42 All other IFs, even those without dedicated provisions, under the potentially applicable provisions provide for acts intended to cause such offences to be sanctioned. The IFSC’s applicable provisions have general language which do not imply similarly.

43 See WR Regulations on Anti-Corruption and Betting in the WR Regulations Handbook (2016) which states under Regulation 6.3 “*it is not necessary that intent, fault, negligence and/or knowing commission of an Anti-Corruption Breach on the Connected Person’s part be demonstrated in order to establish that an Anti-Corruption Breach has been committed*.” Interestingly, neither intent nor negligence is considered necessary to demonstrate in this case.

44 Item 5 under the IBSF’s Code of Ethics.

45 See Article 19, of the FIH Integrity Code (2020).

46 See IFSC’s provisions across the IFSC Statutes (2019) under objectives as well as general obligations making a reference to the IOC 2016 Code in athlete consent forms as present on its website.

47 See sample definition extracted in Sect. 2.2 above.

48 This type of manipulation has been seen in cases such as *Vanessa Vanakorn* v. *FIS,* CAS 2014/A/3832 and 3833, award dated 19 June 2015; there was held to have been manipulation under the applicable FIS provisions, where the CAS overturned the FIS Council (internal FIS appellate body’s decision) finding manipulation under applicable FIS Betting and other Anti-Corruption and Violation Rules, 2009, though at both levels, the issue of there being financial gain to prove an offence under the applicable rules did not arise. Those rules have since been replaced by the FIS Rules on the Prevention of Manipulation of Competitions (2016).

49 Explanatory Report (explanation to Article 3) to the Macolin Convention, 18 September 2014 at p. 10.

50 See Diaconu and Kuhn (2019) in section 4.2 (p. 10) where they cite critique of the approach of criminalization of such acts and the Macolin Convention’s approach therefore as seen in Chappelet (2015), at p. 1263, where it is considered to be beyond the scope of what the Macolin Convention seeks to address.

51 See Section 2.1 of this article, *supra*.

52 Including, but not limited to “*bribes, gains, gifts and other advantages including, without limitation, winnings and/or potential winnings as a result of a wager, excluding prize money, appearance fees or payments by sponsors or under contracts*”—under Article 1.1 of the IOC 2016 Code.

53 See PMC 2.1 under the FINA PMC Rules.

54 Article 18 of the FIFA Disciplinary Code (2019) and Article 29 of the FIFA Code of Ethics (2020) states, “*Anyone who directly or indirectly, by an act or an omission, unlawfully influences or manipulates the course, result or any other aspect of a match and/or competition or conspires or attempts to do so by any means shall be sanctioned... If a player or official engages in behaviour described in paragraph 1, the club or association to which the player or official belongs may be sanctioned ...Persons bound by this Code must cooperate fully with FIFA at all times in its efforts to combat such behaviour and shall therefore immediately and voluntarily report to the secretariat of the Disciplinary Committee any approach in connection with activities and/or information directly or indirectly related to the possible manipulation of a football match or competition as described above. Any breach of this provision shall be sanctioned...(under Disciplinary Code)*.” Corruption and bribery (Article 27) and Misappropriation of Fund (Article 28) are defined independently in the Code of Ethics (2020).

55 Under Section B.4 of the TACP, 2020 as referring to “*anything of value except for money*.” This is used in specific offences under the broader heading of “Corruption Offences” as defined under the TACP, 2020, extracted in Section 2.3 below, sub-sections D.1.d to D.1.g describing manipulation offences in specific, where in the term “*money, benefit or other Consideration*” are used in lieu of “benefit” as ordinarily found across IF regulations.

56 See Appendix—1 (Definitions) at of the ICC Anti-Corruption Code (2020, p. 28)— “*A person acts “for Reward” if he/she arranges or agrees that he/she or some other third party will receive any direct or indirect financial or other benefit for that act (other than official prize money and/or contracted payments under playing, service, endorsement, sponsorship or other such similar contracts)…*.”

57 The IOC 2016 Code (Article 1.4), and consequently many IFs include three categories of persons within “Participants” being—a. athletes or participants in a sports event; b. athlete support personnel being an athlete’s entourage assisting them at sporting functions; c. officials being owners, shareholders, executors and staff of entities organizing and promoting sport/clubs/recognition bodies, or referees, jury or other accredited persons (residual).

58 Per Regulation 6.3, of Regulation 6 (Anti-Corruption and Betting) in the WR Regulations Handbook (2016) “Connected Persons” may commit Anti-Corruption Offences; defined (under Regulation 6.2) as “*International Player, Contract Player, International Match Official, Contract Player Support Personnel, any coach, trainer, selector, health professional, analyst, team official, administrator, owner, shareholder, director, executive, staff member and/or any other person involved with and/or engaged in relation to the Game by a Union or its National Representative Team and shall include any Union/Association/World Rugby panel of Match Officials at International Match and/or Contract Player level, Disciplinary Personnel, any Agent and/or representative of an International Player, Contract Player or Contract Player Support Personnel and/or family member and/or associate of any of the foregoing (to the extent that such family member/associate falls under the jurisdiction of a Union, Rugby Body and/or World Rugby) and/or any other individual or entity involved in the organization, administration and/or promotion of the Game at International Match and/or Contract Player level and/or the training of persons participating in the Game at International Match and/or Contract Player level*.”

59 FIFA’s Disciplinary Code (2019), for example does not have a defined term but merely a list of persons (under Article 3 on the Scope of Application) to whom the Code applies, including the manipulation provision.

60 An example of this is FIBA’s Code of Conduct under the FIBA Internal Regulations (Book 1—November 2020 edn. at p. 22 and 23) where the defined term applicable to manipulation offences is “Basketball Parties” (Items 145–149 on p. 38), which includes all FIBA Divisions, national federations, clubs, cities or states involved in bidding, as well as Participants, Spectators and other “Persons” as defined, whether or not engaged in related activities whether paid or unpaid (p. 25). In turn, a “Participant” for purposes limited to that Code include players, agents, representatives, support personnel, other persons named in rosters, commissioners, game officials, miscellaneous persons with responsibilities (statisticians, interpreters, etc.), persons affiliated to FIBA or organizing committees, other persons in attendance at affiliated games. A “Connected Person” would be any person with who such person has a relationship likely to appear to a reasonable person to influence their objectivity, including family, partners or close personal friends. Persons include natural persons or an organization or another entity (bodies corporate, unincorporated associations or partnerships), whether or not with a separate legal identity (p. 24).

61 See, for example, Section I.2.C, of the WS Code of Ethics and Code of Conduct (2019) on p. 4 which lists exhaustively entities to include potentially within the realm of actors who could be liable all possible entities.

62 Rule 3.4 of the IIHF Code of Conduct (2021) includes “*Knowingly assisting, aiding, abetting, attempting, covering up or otherwise being complicit in the commission of any acts or omissions*” as “General Violations” over and above a specific manipulation offence. The IOC 2016 Code includes these elements under Article 2.7.2 which exists to assist in determining by laying out elements relevant (or not relevant) to whether an offence has been made out.

63 Under Annexure 2, para 3 to the ICC’s Anti-Corruption Code for Participants (amended January 1, 2021), extending the definition of “Participants” to those who would not ordinarily fall within it but who “*pose a genuine threat to the integrity of cricket*”; instances of such situations are provided under para 4.

64 For instance, Diaconu et al. (2021) in Section 6.2 (*Ratione Personae*) list across CAS awards, most on appeal from IF decisions and applying their regulations, which parties, including athletes, referees, clubs and their officials (and clubs due to their officials), have been convicted of manipulation.

65 By the FIFA Disciplinary Committee for match-fixing by individual players, banning the club from the 2020–2021 season; see https://www.fifa.com/who-we-are/news/fifa-disciplinary-committee-sanctions-kenyan-club-zoo-fc-for-involvement-in-matc (May 5, 2021).

66 Initially seen in cases such as *Public Joint-Stock Company “Football Club Metalist”* v. *UEFA* and *PAOK FC*, CAS 2013/A/3297 award dated 29 November 2013 under UEFA’s applicable regulations for eligibility under the UEFA Champions League Regulations for the 2013–2014 season—paras 8.35 and 8.36 and *Pakruojo* at paras 1–19 and 26.

67 Section I.3.B (Sanctions Applicable only to Natural Person), I.3.C (Sanctions Applicable to Legal Person) and I.4 (Level of Responsibility) of the WS Code of Conduct and Code of Ethics (2019, pp. 5 and 6).

68 For instance, both the IOC 2016 Code under Article 2.2 and other definitions (for example, FINA’s which states “…*with the goal of obtaining a Benefit for oneself or for others*” —under PMC 3.2 in FINA’s PMC Rules (2016)).

69 FIFA’s definition of manipulation under Article 18 of the Disciplinary Code (2019), for example, as mentioned above, does away with the element of an actor having the aim of obtaining “benefit” whether for themselves for another, and is just based on the positive act of manipulation/attempting to do so—which implicitly would include acts whether or not done for one’s own or for another’s benefit.

70 WS’s Code of Conduct and Code of Ethics (2019) which includes it’s defined manipulation offence and which incorporates the IOC 2016 Code by reference, applies to “*apply to both everyday activities as well as to each and every competition, championship, cup and to any other event either staged or sanctioned by World Skate*.” —Section I.2.A, p. 3.

71 The FINA PMC Rules (2016) state, in PMC 1.1. that they are applicable to only “FINA Competitions” and laws of Major Event Organizations are deferred to for Competitions of “Major Events Organizations” as defined (PMC 2.2 and 2.4 respectively) in absence of which the FINA PMC Rules apply i.e. a limited set of contexts.

72 See for example, Rule 12 of the IAAF’s Manipulation of Sport Competition Rules (2019).

73 The two IFs where this is not the case are IWF (though definitions are the same as the IOC 2016 Code, the titles of the respective section state that they are specific to betting—Article 2 of the IWF Guidelines on Competition Fixing, 2015) and IFSC (manipulation could be brought within wider misconduct offences, or for bringing the sport into disrepute, or if betting related, under specific offences—see Article 5 bis and Bylaw to Article 10 of the IFSC Statutes (2019); under both sets of regulations the inclusion of non-betting related manipulation is thus unclear.

74 For instance, for the IFs following the IOC 2016 Code, under Article 2.1 the definition of prohibited forms of “Betting” usually precedes that of manipulation and states that “*Betting in relation either: a. to a Competition in which the Participant is directly participating; or b. to the Participant’s sport; or c. to any event of a multisport Competition in which he/she is a participant*.” constitutes an offence.

75 This is, for example, the case in the IOC 2016 Code and consequently the federations which have adopted it, as well as the regulations within the Code of Ethics for the Tokyo 2020 Regulations, which incorporates the code.

76 Examples include IWF (where the IWF Guidelines on Competition Fixing applicable regulation title mentions that it is in the “Specific Context of Betting”) and the IFSC (whose Disciplinary Appeals Rules, 2019 talk of “betting and gambling offences” (p. 34), as well as Article 10 of its statutes defining “illegal and irregular betting”. WTF and UIPM also contain “Betting” within the title of their respective regulations.

77 For example, Article 5 bis of the IFSC Statutes (2019) states that one of the IFSC’s objectives is “*To fight against competition manipulation*” and “*To fight against irregular or illegal betting or gambling*”. Bylaw to Article 10 of the IFSC Statutes defines the violation of “*Illegal and irregular betting*”, but not manipulation itself. Article 38 refers to the IFSC Disciplinary and Appeals Rules (2007, updated 2019) which contains an independent schedule of offences, which includes, in addition to the offence above in the Statutes, a few other manipulation related offences, but does not *per se* have one overarching definition of fixing or manipulation.

78 Article D.1 of the ITF’s TACP, 2020.

79 See, for example, the robust definition of “Prohibited Betting” under Regulation 6 on Anti-Corruption and Betting in the WR Regulations Handbook (2016).

80 See, for example, Rule 5 of the IAAF Manipulation of Sport Competition Rules (2019).

81 See, for example the ICF Statutes which incorporate the IOC 2016 Code (*supra* note 9) and thereby the offences connected to reporting under Article 2.5 thereunder.

82 See Article 2.5, titled “Idealism” under Article 2 dealing with Fundamental Ethical Principles—two offences (Article 2.5.1 on all types of betting or wagering, and Article 2.5.2 on general manipulation) are provided for, and Article 3 addresses specifically officials’ need to be objective in judging.

83 Article 12 of the ISU Code of Ethics (2018).

84 See “Integrity”, UEFA, available at https://www.uefa.com/insideuefa/protecting-the-game/integrity/#legalframework (April 10, 2021); the eminence of the regulations and their evolution has been discussed in detail in Garcia Silvero (2018, p. 6).

85 Article 12 of the UEFA Disciplinary Regulations (2020 edn.), covering manipulation and betting, the result and course of an event, for gaining an advantage for themselves or a third person, sharing insider information, reporting obligations *inter alia*, pp. 14 and 15.

86 Article 8 “Responsibility” within the UEFA Disciplinary Regulations (2020 edn.)

87 Articles 15 and 16 of the UEFA Statutes (1993 edn., “1993 UEFA Statutes”).

88 “Unforeseen circumstances” under Article 28 of the 1993 UEFA Statutes; the case of *Royal Sporting Club Anderlecht* v. *UEFA*, TAS 98/185 was the appeal of a UEFA Executive Council decision to the CAS, who then decided that judicial functions could not be undertaken by Executive Bodies in this manner.

89 Garcia Silvero (2018, p. 9).

90 Then Article 50(3) on “Non-Admission” of the UEFA Statutes (amended in 2007, valid to date), states “*The admission to a UEFA competition of a Member Association or club directly or indirectly involved in any activity aimed at arranging or influencing the outcome of a match at national or international level can be refused with immediate effect, without prejudice to any possible disciplinary measures*”. This is reflected in the regulations of each of UEFA’s leagues (Champions League, Europa League and the Women’s Champions League) under Articles 4.02 and 4.03 of each of their regulations. Article 4.02 states, *inter alia*, that “*If, on the basis of all the factual circumstances and information available to UEFA, UEFA concludes to its comfortable satisfaction that a club has been directly and/or indirectly involved, since the entry into force of Article 50(3) of the UEFA Statutes, i.e. 27 April 2007, in any activity aimed at arranging or influencing the outcome of a match at national or international level, UEFA will declare such club ineligible to participate in the competition. Such ineligibility is effective only for one football season…*”

91 Articles 4.03 of each regulations state “*In addition to the administrative measure of declaring a club ineligible as provided for in* *Paragraph 4.02, the UEFA Organs for the Administration of Justice can, if the circumstances so justify, also take disciplinary measures in accordance with the UEFA Disciplinary Regulations*.”

92 IOC Guidelines for Sports organizations the Sanctioning Competition Manipulation (2018).

93 Articles 2.2 and 2.3 of the IOC 2016 Code.

94 IOC Sanctioning Guidelines, p. 13.

95 See types of sanctions under Section I.3.A of the WS Code of Ethics and Code of Conduct (2019, pp. 4 and 5).

96 These could include suspensions, the decision to video record a competition, the decision to change the referee or judges just prior to the commencement of a competition, and in certain cases where there is particular risk to the reputation of the sport. Further, they may or may not be counted toward the final sanction issued—see Article 4.1 to 4.3 of the WKF Code on Prevention of Manipulation of Competitions (2016). Similarly, FIFA’s Disciplinary Code (2020—Article 48) and Code of Ethics (2020—Articles 84 and 85), provide that “*necessary to ensure the proper administration of justice, to maintain sporting discipline or to avoid irreparable harm, or for reasons of safety and security*” even without hearing parties, and subject to a maximum 90 days, which may be extended to aid investigations.

97 See, for example, Section I.3.B and I.3.C of the same WS Code of Ethics and Code of Conduct (2019, p. 4).

98 Rule 8.3 (p. 9) of the FIS Rules on the Prevention of Manipulation of Competitions on “Financial Sanctions” stating the fine is to be based on “*maximum of the value of any Benefit received by the Participant, directly or indirectly, as a result of his breach of these Rules*.” A similar approach is seen in PMC 5.1.1 of FINA’s PMC Rules (2016).

99 Under FIBA Internal Regulations (Book 1, Nov 2020 edn., p. 41), the fines possible to imposed were up to CHF 300,000 for entities and CHF 100,000 for natural persons for manipulation offences—see Items 164.c and 165.c; the FIE (under Appendix 1 to Chapter 12, i.e. Appendix 1 to the FIE Ethical Code: Betting and Anti-Corruption Rules of FIE Statutes (2015, p. 51) under Article 7.1.4.f (p. 34) being between CHF 125 and CHF 12,500 for individuals and CHF 225 and CHF 22,500 for “entities”.

100 See for example limits based on seriousness of the offence under section 164.14 of the FEI’s General Regulations (24th edn., Jan 2020, p. 39) which follow a gradation system of low end (CHF 1000 to CHF 1500), mid-range (CHF 2000 to CHF 3000), top-end (CHF 5,000 to CHF 10,000) to max (CHF 15,000); and FIH (ranging between CHF 500 and CHF 20,000).

101 Article 25 of the FIG Code of Discipline (2019) which specifies sanctions, and is referred to under Article 5 of the FIG Code of Conduct (2019) leaves it to the discretion of the Disciplinary Authority; under Article 18.1 of the FIFA Disciplinary Code, 2019 a minimum fine of CHF 100,000, is prescribed without stating a maximum.

102 Under the same Article 18.1 of the FIFA Disciplinary Code, 2019 the fine is prescribed but the primary sanction remains a ban from football related activities for a minimum of five years (and/or a fine).

103 Fines should be staggered on this basis—under Article 29 of the FIH Integrity Code (2018) of which Article 9 is the FIH “Anti-Corruption Code” which includes betting and manipulation offences, to which common sanctions as present in the rest of the FIH Integrity Code apply.

104 Proportionality of sanctions issued for disciplinary offences given these factors have been questioned in the past by practitioners and academics; see (in manipulation context) Diaconu et al. (2021) and (in the context of doping) Rigozzi and Quinn (2019); in certain cases, IFs provide guidance on life-bans—FIH, for example, states that life-bans are only to be issued where “*particularly serious breach*” has occurred—Article 27.1.8 of the FIH Integrity Code (2018).

105 ITTF’s Handbook (2020) under Regulation 6.9 (p. 182) tackles manipulation offences with limits on ineligibility based on the type of offence (betting, manipulation, corrupt conduct, inside information, or other violations) ranging from two years to four years (Article 6.9.7.3.2, p. 190).

106 Article 7.1.j of the FIFA Code of Ethics (2019) allows for the option of a “*ban from taking part in all football-related activities*” as a disciplinary sanction.

107 Similarly, Articles 7.1.h and 7.1.i of the same FIFA Code of Ethics (2019) allow for the issuance of a limited ban from dressing rooms/the substitute’s bench or from entering a stadium.

108 Section I.3.A of the WS Code of Ethics and Code of Conduct (2019, pp. 4 and 5).

109 PMC 5.1.3 of the FINA PMC Rules (2016) provides for “Education and Rehabilitation” as a sanctions and  a precondition to eligibility to participate after a period of ineligibility issued.

110 Article 7.1.f of the FIFA Code of Ethics (2019) provides “social work” as a potential disciplinary sanction.

111 Article 9.1.9 of Chapter E on Procedures for the Investigation and Prosecution of Violations of the IBU Integrity Code as part of the IBU’s Integrity Code (2019) under which manipulation offences are brought, allows the Disciplinary Tribunal to prescribe “mandatory education sessions” as a potential sanction for offences.

112 Article 5.1 of the WKF Code on the Prevention of Manipulation of Competitions (2016) lists “education” as one of the possible sanctions that might be awarded under the code and the Disciplinary and Ethics Code (2016).

113 Community football service under Articles 6.1.r (associations and clubs) and 6.2.h (individuals) of the UEFA Disciplinary Regulations (2020 edn.).

114 See Diaconu et al. (2021) under Section 8 on Sanctions, where it is observed that most CAS awards confirm findings of federation bodies, and particularly in manipulation offences.

115 See, for example, the award of the CAS in *Butt*, *infra* note 148 at paras 56–57, 60, 66–69 and 74 –75.

116 See Diaconu et al. (2021) under Section 8 on Sanctions.

117 See FIFA sanctions available at https://www.fifa.com/who-we-are/legal/integrity/leading-cases/, including *Mwamelo*, *infra* note 124 (15 year ban), with 9 other life bans in 2019.

118 See ITIA sanctions available at https://itia.tennis/news/sanctions/—April 27, 2021. The CAS also upheld the sanction awarded to Juan Carlos Sanchez of eight years of suspension with a fine of USD 12,500 and legal costs of CHF 3000—https://itia.tennis/news/sanctions/cas-upholds-sanction-for-juan-carlos-saez/ (April 27, 2021). Prior bans include 2020 include the life-ban awarded to Ukrainian player Mr. Stanislav Poplavsky for multiple fixing instances between 2015 and 2019—https://itia.tennis/news/sanctions/stanislav-poplavskyy-receives-lifetime-ban-tennis/ (April 27, 2021) and Algerian player Aymen Ikhlef for ten breaches of the TACP (2020) being four instances of match fixing, two instances of soliciting other players not to use their best efforts, three instances of failure to report a corrupt approach and one charge of failing to cooperate with the TIU investigation—https://www.itia.tennis/news/sanctions/algerian-tennis-player-banned-life/ (April 27, 2021).

119 United Arab Emirates’ captain Mohammed Naveed and player Shaiman Anwar Butt were awarded eight year bans from all forms of cricket in March 2021—https://www.icc-cricket.com/media-releases/2069768 (April 27, 20210), with the teammate Qadeer Ahmed Khan (banned for five years); similarly, Sri Lanka’s Dilhara Lokuhettige (eight years), Nuwan Zoysa (six years) were also sanctioned in April 2021 (see https://www.icc-cricket.com/media-releases/—April 27, 2021). This is in addition to the high profile case of Zimbabwean Heath Streak (discussed below in *infra* note 130).

120 See Pavitt (2021)

121 The World Anti-Doping Agency’s World Anti-Doping Code (2021), for instance, provides a ranges and factors relevant to sanctioning across the offences.

122 See ECtHR, *Vinter and Others v. the United Kingdom*, judgment (Grand Chamber) of 9 July 2013, §§ 119–122.

123 For example, as concluded in Rigozzi (2020), p. 128.

124 See FIFA Disciplinary and Regulatory Commission decision of Mr. Boniface Mwamelo (Football Association of Zambia) under the 2009 FIFA Code of Ethics, where FIFA’s life ban and fine and CHF 10,000 fine were reduced to a 15 year ban and CHF 10,000 fine by CAS (in July 2020) for engaging with convicted match-fixers over email with the aim of manipulating Zambia U-23 matches for financial gains through betting; *Boniface Mwomelo* v. *FIFA*, CAS 2019/A/6220 (“Mwamelo”); see similar ban period reduction by the CAS (award of March 2020) in *Sidio Jose Mugadza* v. *FIFA*, CAS 2019/A/9219 from 15 to seven years.

125 See for example, Article 27.2.2.1 and 27.2.2.2 of the FIH Integrity Code (2018). Article 27.3 therein also stated that where “*more than one breach has been committed, the sanction will be based on the most serious breach, and increased as appropriate depending on the specific circumstances.*”

126 Article 27.1.7 of the same FIH Integrity Code (2018) as specified particularly for determining period of ineligibility.

127 Under Item 4 “Level of Responsibility” which describes factors to be considered while issuing sanctions under the WS Code of Conduct and Code of Ethics (2019, p. 6).

128 The IFSC Disciplinary and Appeals Rules (2019) under Article 20.1 and 20.2 provide a specific time period for bans and amount for fines for types of offences (Items 8, 9 and 11). Certain subjective unspecified “*exceptional circumstances*” might be considered when sanctioning as listed under Article 23.

129 In addition to FIH above, see also, for example the Article 9 of the FIFA Code of Ethics (2020); such factors include “*nature of the offence; the substantial interest in deterring similar misconduct; the offender’s assistance to and cooperation with the Ethics Committee; the motive; the circumstances; the degree of the offender’s guilt; the extent to which the offender accepts responsibility, and whether the person mitigated his guilt by returning the advantage received, where applicable. 2. In case of mitigating circumstances, and if deemed appropriate taking into account all circumstances of the case, the Ethics Committee may go below the minimum sanction and/or decide to hand down alternative sanctions*…” Under the FIFA Disciplinary Code (2019), Article 24 specifies that “*The judicial body determines the type and extent of the disciplinary measures to be imposed in accordance with the objective and subjective elements of the offence, taking into account both aggravating and mitigating circumstances… …When determining the disciplinary measure, the judicial body shall take into account all relevant factors of the case, including any assistance of and substantial cooperation by the offender in uncovering or establishing a breach of any FIFA rule, the circumstances and the degree of the offender’s guilt and any other relevant circumstances. …In exercising its discretionary powers, the relevant FIFA judicial body may scale down the disciplinary measure to be imposed or even dispense with it entirely*.”

130 ICC Anti-Corruption Code—in the matter of proceedings between the ICC and Mr. Heath Streak, dated March 29, 2021 available at https://resources.pulse.icc-cricket.com/ICC/document/2021/04/14/e06b37f8-65cb-4d0b-811b-d7bc0bbe3c2a/Decision-of-the-ICC-28-March-2021.pdf (April 5, 2021) at para. 33.

131 See *supra* note 124 on CAS alteration (reducing) of sanctions in FIFA DRC decisions; in *Mwamelo* mitigating factors included comparatively small amounts, singular attempt at manipulation, a first offence, incitement by a third party, and the fact that a less harsh sentence could achieve the same purpose.

132 Item 4.A “‘Level of Responsibility” under the WS Code of Conduct and Code of Ethics (2019, p. 5 and 6) categorizes the levels of responsibility into these four categories.

133 See WBF Sentencing Guidelines (2015), where factors specific to the sport of bridge, such as collusive cheating, obtaining information related to hands, and inadvertent cheating are all sanctioned in a specific manner under the guidelines. Discounted sentences are also similarly provided for.

134 In light of the aforementioned numerous life-bans issued in 2021 (Sect. 3.2.2 above), the recent decision regarding a basketball player in Lithuania who admitted to instances of fixing in eight lost matches, considering severe repentance, no prior record and assessment of lack of likelihood to commit further crimes could be considered  significantly less harsh—https://www.sportsintegrityinitiative.com/sports-integrity-briefs-19-may-2021/ (May 19, 2021).

135 See generally, Swiss Federal Tribunal pronouncement in *Gundel* v. *FEI*, SFT 119 II 271, decision dated 15 March 1993.

136 *Johannes Eder* v. *Ski Austria*, CAS 2006/A/1102, award dated 13 November 2006 where CAS made the distinction between civil sanctions issued by a sporting body and criminal sanctions by a state (para. 52).

137 See *AEK Athens and Slavia Prague* v. *UEFA*, CAS 98/2000, award dated 20 August 1999.

138 This has been observed multiple times, though an argument could be made on whether further thought should be given to this principle’s operation across various levels of adjudication in a sport and parallel to criminal proceedings—see Diaconu et al. (2021) at p. 44; the same concerns have also been raised previously in Palermo and Williams (2018, p. 21).

139 See Valloni and Pachmann (2013).

140 Leading cases prosecuted by FIFA are listed here with the relevant decisions—https://www.fifa.com/who-we-are/legal/integrity/leading-cases/ (April 8, 2021). Most recently the decision issued in September, 2020 awarding a life-time ban and fine of CHF 100,000 to Marco Antonio Trovato Villalba, President of Olimpia, a Paraguayan club for manipulation and failure to cooperate under Article 18 and Article 20 respectively of the FIFA Disciplinary Code (2019) for two matches in 2018 and 2019—see https://www.fifa.com/who-we-are/news/the-fifa-disciplinary-committee-sanctions-marco-antonio-trovato-villalba-for-mat (April 8, 2021). The FIFA Appeal Committee had 3 decisions connected to manipulation in 2019/2020 (reduced number due to Covid-19)—see FIFA Disciplinary and Ethics Report 2019/2020, available at https://resources.fifa.com/image/upload/disciplinary-ethics-report-2019-20.pdf?cloudid=hnuvyrpsxcu5lllv6sxy (April 9, 2021).

141 The cases arising from UEFA are listed chronologically under “Integrity” here, available here https://www.uefa.com/insideuefa/protecting-the-game/integrity/#cases (April 5, 2021).

142 See Section 5 of Diaconu et al. (2021) which lists each case before the CAS.

143 See ITIA, *Sanctions*, available at https://www.itia.tennis/rules/sanctions/ (April 7, 2021). Note—the page mentions that this is the list of athletes currently serving sanctions.

144 ITF, ATP, WTA and the Grand Slam Committees, albeit not all “IFs” strictly.

145 Life bans—*Daniel Köllerer* v. *Association of Tennis Professionals* (“ATP”)*, Women’s Tennis Association, International Tennis Federation and Grand Slam Committee*, CAS 2011/A/2490, award dated 23 March 2012 and *David Savic* v. *Professional Tennis Integrity Officers*, CAS 2011/A/2621, award dated 5 September 2012; ineligibility—*Guillermo Olaso de la Rica* v. *Tennis Integrity Unit*, CAS 2014/A/3467, award dated 30 September 2014.

146 See ICC, *ICC Anti-Corruption Code: Recent ICC Decisions* available at https://www.icc-cricket.com/about/integrity/anti-corruption/acu-publications (April 14, 2021)

147 Sanctions for offences under Articles 2.3.2, 2.3.3, 2.4.2, 2.4.4 and 2.4.7 of the ICC_ACU decision on Mr. Heath Streak (*supra* note 130); sanctions for offences under Articles 2.1.1, 2.1.4 and 2.4.4 of the same code—in proceedings between the ICC and Mr. Dilhara Lokuhettige available at https://resources.pulse.icc-cricket.com/ICC/document/2021/04/19/d1431a99-eb58-45bb-be30-90d2cdc5f874/ICC-v-Lokuhettige-Decision-on-Sanctions-7-April-2021-redacted-PDF.pdf (April 10, 2021).

148 *Mohammad Asif* v. *International Cricket Council*, CAS 2011/A/2362 (“Asif”), award dated 17 April 2013 and *Salman Butt* v. *ICC*, CAS 2011/A/2364, award dated 17 April 2013 (“Butt”) both had pending English Criminal Court decisions being issued along-side which factored in the parallel proceedings into sentencing—see paras 70 and 71 in *Asif.*

149 See Mavromati and Reeb (2015, p. 379 onward).

150 Diaconu et al. (2021) at p. 43.

151 Article 190(2) provides grounds including improper arbitrator appointment or constitution of the arbitral tribunal, wrong acceptance or declination of jurisdiction, decision beyond claims or failure to decide a claim, violation of principle of equal treatment or failure to be heard, and incompatibility of the award with Swiss public policy.

152 This approach is adopted by FIS, and among the summer federations, by IGF, IWF, WT and ITTF.

153 FINA (certain initial procedure, and sanctions are common to all offences), WA (common bodies responsible for prosecution and sanctions common to all offences), AIBA, FEI, FIE, WKF (for these four, certain common procedural regulations are present in applicable disciplinary regulations while others are in manipulation specific regulations), ITF (TACP applicable except on the admissibility of certain evidence), WR (certain adjudication regulations which are common with other offences as well, save for when there is a conflict) and ISA (specific regulations for certain processes, sanctions issued under an independent rulebook).

154 See, for example, IAAF’s Reporting, Investigation and Prosecution Rules—Non-Doping (2019) and Disciplinary Tribunal Rules (2019).

155 FIH’s Integrity Code (2018) provides, for example, that it is a *“valid defence to a charge of a breach of this Integrity Code if the Covered Person adduces sufficient credible evidence to prove, on balance of probabilities, that genuine and powerful reasons exist (or existed) to justify his conduct objectively, taking into account all the relevant circumstances. For this purpose, the right to invoke the privilege against self-incrimination is deemed to have been waived by each Covered Person*.”

156 See, for example, Rule 4 of the Disciplinary Code of the FIE (Chapter 7, in FIE’s Statutes, 2015).

157 See *Sport Lisboa and Benfica Futebol SAD* v. *UEFA and FC Porto Futebol SAD* and *Vitória Sport Clube de Guimarães* v. *UEFA and FC Porto Futebol SAD*, CAS 2008/A/1583 and 1584, award dated 15 July 2008 at paras 39–41.

158 See Rules 6.2 and 6.3 of the Disciplinary Code of the FIE (Chapter 7, in FIE’s Statutes, 2015).

159 See, for example, Rule 5.2 under Disciplinary Code of the FIE (Chapter 7, in FIE’s Statutes, 2015).

160 Article 157(d) under Appendix G (FEI Code on Prevention of Manipulation of Competitions) to FEI General Regulations (2020).

161 Article 163 Appendix G (FEI Code on Prevention of Manipulation of Competitions) to FEI General Regulations, (2020).

162 These factors have been noted across cases, for example in *Mr. Oleg Oriekhov* v. *UEFA*, CAS 2010/A/2172 award dated 18 January 2011.

163 Further, even in other major event regulations, there is seen a heightened specific focus on certain offences, leaving out corruption; most recently, see the governing Regulations issued for the 30th Winter Universiade 2021 which was to have been held in Lucerne in December 2021, available at https://www.fisu.net/medias/fichiers/regulations_wu2021_lucerne_20200120.pdf, April 10, 2021).

164 See, for example, Sir Richard McLaren’s opinion in *Match-fixing, not doping, poses greatest risk to sport*, Reuters (2015).

165 See for examples issues discussed at forums such as the CSCF Foundation for Sport Integrity and IntegriSport Erasmus+ project which ran from 2019 to 2020—see https://cscfsport.com/media-statement-project-closing-conference-sports-manipulations-and-criminal-activities-impacting-sport/ (April 10, 2021).

166 Most recent INTERPOL Bi-Weekly Bulletins have referred to an eSports manipulation or betting related investigation/alert; for example, sanctions were issued by ESIC to 35 persons in Australia earlier this year—INTERPOL Bi-weekly Bulletin Jan- Feb, 2021.

167 See Sports Law Yearbook (2015-6)
